# Learning Cortical Parcellations Using Graph Neural Networks

**DOI:** 10.3389/fnins.2021.797500

**Published:** 2021-12-24

**Authors:** Kristian M. Eschenburg, Thomas J. Grabowski, David R. Haynor

**Affiliations:** ^1^Department of Bioengineering, University of Washington, Seattle, WA, United States; ^2^Integrated Brain Imaging Center, University of Washington Medical Center, Seattle, WA, United States; ^3^Department of Radiology, University of Washington Medical Center, Seattle, WA, United States; ^4^Department of Neurology, University of Washington Medical Center, Seattle, WA, United States

**Keywords:** graph neural network, parcellation, functional connectivity, representation learning, segmentation, brain, human

## Abstract

Deep learning has been applied to magnetic resonance imaging (MRI) for a variety of purposes, ranging from the acceleration of image acquisition and image denoising to tissue segmentation and disease diagnosis. Convolutional neural networks have been particularly useful for analyzing MRI data due to the regularly sampled spatial and temporal nature of the data. However, advances in the field of brain imaging have led to network- and surface-based analyses that are often better represented in the graph domain. In this analysis, we propose a general purpose cortical segmentation method that, given resting-state connectivity features readily computed during conventional MRI pre-processing and a set of corresponding training labels, can generate cortical parcellations for new MRI data. We applied recent advances in the field of graph neural networks to the problem of cortical surface segmentation, using resting-state connectivity to learn discrete maps of the human neocortex. We found that graph neural networks accurately learn low-dimensional representations of functional brain connectivity that can be naturally extended to map the cortices of new datasets. After optimizing over algorithm type, network architecture, and training features, our approach yielded mean classification accuracies of 79.91% relative to a previously published parcellation. We describe how some hyperparameter choices including training and testing data duration, network architecture, and algorithm choice affect model performance.

## 1. Introduction

Neural network approaches such as multi-layer feed-forward networks have been applied to a wide variety of tasks in medical imaging, ranging from disease classification to tissue segmentation. However, these networks do not always take into account the true spatial relationships between data points. Convolutional neural network approaches, such as those applied to static images or dynamic video streams, learn translationally-invariant, multidimensional kernel filters over the data domain. Both these methods assume that the data is sampled regularly in space, allowing convolution and pooling of information from fixed neighborhood topologies. However, real-world data, such as graph-structured data, is often sampled on irregular domains. Data sampled from graph domains often contains non-uniform topology—individual data points can vary in their neighborhood structure, and notions of direction (e.g., up, down, left, right) do not generalize well to graphs. This makes learning filters to process graph-structured data very difficult with conventional neural network approaches.

Graph neural networks are a class of neural network models that operate on data distributed over a graph domain. Data are sampled from a graph with an explicit structure defined by a set of nodes and edges. These models have been shown to be useful for graph and node classification tasks, along with learning generative models of data distributed over graphs (Kipf and Welling, 2016b; Hamilton et al., 2017; Zhao et al., 2019; Zeng et al., 2020). Graph convolution networks (GCN), proposed in Defferrard et al. (2016), generalized the idea of convolutional networks on grid-like data to data distributed over irregular domains by applying Chebyshev polynomial approximations of spectral filters to graph data. Graph attention networks (GAT) are based on the idea of an attention function, a learned global function that selectively aggregates information across node neighborhoods. The attention function maps a query and set of key-value pairs to an output (Vaswani et al., 2017). The output is defined as a weighted sum of the values, where weights are computed using some similarity or kernel function of the key-value pairs.

It is believed that biological signals distributed over the cortical manifold are locally stationary. Given a small cortical patch, voxels sampled from the patch will display similar functional and structural connectivity patterns, cortical thickness and myelin density measures, and gene expression profiles, among various other signals (Glasser and van Essen, 2011; Amunts et al., 2020; Wagstyl et al., 2020). Prior studies have attempted to delineate and map the cortex by identifying contiguous cortical subregions that are characterized by relative uniformity of these signals (Blumensath et al., 2013; Arslan et al., 2015; Baldassano et al., 2015; Gordon et al., 2016). This work is based on the fundamental idea that contiguous regions of the cortex with similar connectivity and histological properties will tend to function as coherent units. Biological signals distributed over the cortex exhibit local but not global stationarity, so any attempt to parcellate the cortex must take both properties into account.

Most brain imaging studies utilize cortical atlases—template maps of the cortex that can be deformed and mapped to individual subjects' brains—to discretize the cortical manifold and simplify downstream analyses (Fischl et al., 2004; Bullmore and Sporns, 2012). However, it remains an open question how to “apply” existing cortical maps to unmapped data. A recent study identified considerable variability in the size, topological organization, and existence of cortical areas defined by functional connectivity across individuals, raising the question of how best to utilize the biological properties of any given unmapped dataset to drive the application of a cortical atlas to this new data (Glasser et al., 2016).

Here, we developed an approach to perform cortical segmentation—a node classification problem—using graph neural networks. The cerebral cortex is often represented as a folded sheet, and a usable parcellation approach must be applicable to this sort of data. Neural networks can be extended to account for non-stationarity in MRI volumes by incorporating 3D-volumetric convolution kernels. However, these approaches are not easily applied to data distributed over 2-D manifolds like the cortical surface. Additionally, more recent large-scale studies interpolate neurological signals, like cortical activation patterns or various histological scalar measures, onto the cortical manifold to mitigate the potential for mixing signals from anatomically close yet geodesically distant cortical regions, e.g., across sulci (Yeo et al., 2011; Glasser et al., 2013). These studies could also benefit from methods that operate directly on graphs.

With the growth of large-scale open-source brain imaging databases [ADNI (Petersen et al., 2010), ABCD (Hagler et al., 2019), HCP (Glasser et al., 2013)], neuroscientists now have access to high-quality data that can be used for training models that can then be applied to new datasets. We leveraged the statistical properties of these high-quality datasets to inform the segmentation of new data using multiple variants of graph neural networks. We considered graph convolution networks and two variants of graph attention networks: standard attention networks (Velickovic et al., 2018), and attention networks with adaptive network depth weighting (a.k.a. jumping-knowledge networks, Xu et al., 2018). We examined how algorithm choice and network parameterization affect cortical segmentation performance. We trained our classification models on high-quality open-source imaging data, and tested them on two datasets with unique spatial and temporal resolutions and different pre-processing pipelines. Other methods have been proposed for delineating the cortex using various registration (Fischl et al., 2004; Robinson et al., 2018), neural network (Hacker et al., 2013; Glasser et al., 2016), label fusion (Asman and Landman, 2012, 2014; Liu et al., 2016), and even graph neural network approaches (Cucurull et al., 2018; Gopinath et al., 2019). To the best of our knowledge, this is the first attempt to examine the performance of common variants of graph neural networks in a whole-brain cortical classification setting and explore their ability to generalize to new datasets using functional magnetic resonance imaging (fMRI). While other studies have proposed the use of graph neural networks to delineate cortical areas, these studies did not perform in-depth analyses on how network architecture, algorithm parameter choices, feature type, and training and testing data parameters impact the predicted cortical maps (Cucurull et al., 2018; Gopinath et al., 2019). To this end, we studied how each of these different variables impacts model performance and prediction reliability.

## 2. Background

### 2.1. Graph Convolution Networks

Convolution filters over graphs using spectral graph theory were introduced by Defferrard et al. (2016). For a graph *G* = (*V, E*) with *N* nodes and symmetric normalized graph Laplacian, *L*, define the eigendecomposition of *L* = *U*Λ*U*^*T*^, where the columns of *U* are the spectral eigenfunctions of *G*. Given a graph signal *x* ∈ ℝ^*N*^ distributed over *G*, the graph Fourier transform of *x* is defined as $\tilde{x}=U^{T}x$, and its inverse graph Fourier transform as $x=U\tilde{x}$. Graph filtering of *x* is then defined as $g_{\theta }\left(L\right)x=Ug_{\theta }\left(\Lambda \right)U^{T}x$, where *g*_θ_ is an arbitrary function of the eigenvalues.

Because these filters are not localized in space, Defferrard et al. (2016) proposed to use a Chebyshev polynomial approximation to learn spatially localized filters directly from the Laplacian, reducing the filtering operation of a *x* to


(1) $$\begin{array}{l} g_{\theta }\left(L\right)x=\overset{K-1}{\underset{k=0}{\sum }}\theta_{k}T_{k}\left(L\right)x \end{array}$$

where *T*_*k*_(*L*) and is the *k*-th polynomial and θ_*k*_ the *k*-th learnable Chebyshev coefficient. The polynomial order, *K*, determines the local spatial extent of the filter. If two nodes *i* and *j* are more than *K* hops apart, the filter value *g*_θ_(*L*)_*i, j*_ = 0.

**Figure 1:**
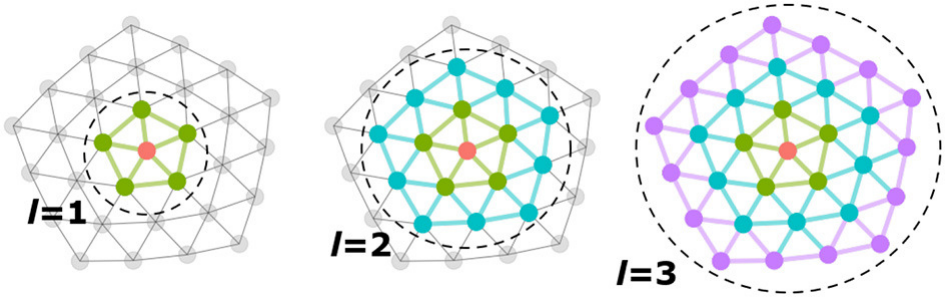
Each layer, *l*, implicitly aggregates more distant neighborhood signals into a node update. The first layer aggregates information over immediately adjacent neighbors, while the second, third, etc. layers incorporate signals from increasingly larger neighborhoods.

In Kipf and Welling (2016a), the polynomial order is set to *K* = 1 so that the spatial extent of the filter is limited to directly adjacent nodes and only one coefficient weight is learned per feature component in each layer of the network. Given $H^{l}\in {\mathbb{R}}^{N\times k_{l}}$, the input feature matrix for layer *l*, the model learns *k*_*l*_ Chebyshev coefficients, in addition to any additional mixing weights. The model incorporates signals from the *l*-ring neighborhood into the update of a node—each layer implicitly aggregates over a larger neighborhood than the previous layer (Figure 1).

### 2.2. Graph Attention Networks

Whereas graph convolution networks uniformly aggregate local neighborhood signals, attention networks learn optimized weights for each node neighbor using an attention mechanism. Assume we have data distributed over a graph with *N* nodes. Inputs to the network are characterized by matrix *X* ∈ ℝ^*N*×*F*^, where *F* is the number of features. Assume that at any given layer, the inputs to layer *l* are represented as $H^{l}\in {\mathbb{R}}^{N\times k_{l}}$, where *H*^0^ = *X*. We define the immediate neighborhood of node *i* as $N_{i}$. For two vectors $\vec{n},\vec{p}\in R^{k}$, we define their feature-wise concatenation as *n*‖*p* ∈ *R*^2*k*^. In Velickovic et al. (2018), the attention paid by node *i* to node $j\in N_{i}$ at layer *l* is computed using a single-layer perceptron as


(2) $$\alpha_{i,j}=\sigma ({\vec{a}}^{T}(W^{l}\vec{h_{i}^{l}}\|W^{l}\vec{h_{j}^{l}}))$$

where σ is a fixed non-linearity, $W^{l}\in {\mathbb{R}}^{k_{l+1}\times k_{l}}$ is a learned layer-specific global linear projection matrix and $\vec{a}$, the attention function, is also learned. The attention weights for $j\in N_{i}$ are then normalized by a softmax operation. To update the features of node *i* at the (*l* + 1)-st layer, we compute the weighted sum over the neighborhood $N_{i}$ with weights defined by the normalized attentions.

Velickovic et al. (2018) propose an ensemble (“multi-head”) attention mechanism, such that, for each layer, *M* different attention functions are learned, each with their own weight vector ${\vec{a}}_{m}^{l}$. The outputs of each attention head are concatenated feature-wise. In the last layer, the number of hidden channels is the number of output classes, *C*—rather than concatenating across attention heads, the outputs of all attention heads are averaged to generate the final network output.

### 2.3. Jumping-Knowledge Networks

While graph neural networks have been instrumental in applying principles of deep learning to graph-structured domains, they are not without pitfalls (Kipf and Welling, 2016a; Velickovic et al., 2018; Xu et al., 2018; Wang et al., 2019). Graph neural networks are prone to over-fitting of model parameters and over-smoothing of learned embeddings as network depth increases (Wang et al., 2019). One approach to alleviate this over-smoothing is to adaptively learn optimized network depths for each node in the graph, a method (Xu et al., 2018) describe as “jumping-knowledge networks.”

Suppose we have a network with *L* layers, such that the *l*-th layer embedding $h_{i}^{l}$ for node *i* is learned by incorporating signals from up to *l* hops away from node *i*. The layer aggregation function described by Xu et al. (2018) learns a unique output embedding by optimally combining the embeddings of each hidden layer as


(3) $$\begin{array}{l} y_{i}=\sigma \left(g\left(h_{i}^{1},h_{i}^{2},\ldots ,h_{i}^{L}\right)\right) \end{array}$$

**Figure 2:**
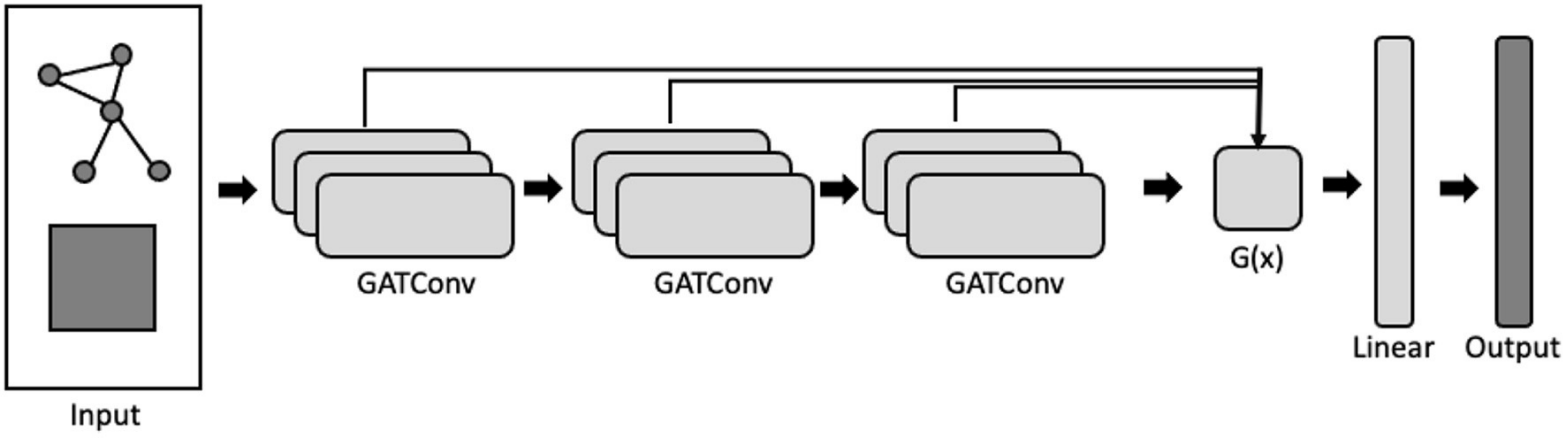
Graph attention network employing a jumping-knowledge mechanism. The network takes as input the graph adjacency structure and the nodewise feature matrix, and outputs a node-by-label logit matrix. Each GATConv block is composed of multiple attention heads. Arrows indicate the direction of processing. Aggregation function, *g*(*x*), which takes as input the embeddings from each GATConv block, learns a convex combination of the layer-wise embeddings.

Xu et al. (2018) propose three permutation-invariant aggregation functions for *g*(*x*): concatenation, max-pooling, and long-short term memory (LSTM) (Hochreiter and Schmidhuber, 1997). The output, *y*, is then passed through a linear feed-forward layer to generate the network probabilities. Concatenation is a *global* aggregator (i.e., the same function is applied to all graph nodes) whereas max-pooling and LSTM both learn node-specific aggregations. Further, by utilizing a bi-directional LSTM layer, jumping-knowledge networks learn layer-specific attention weights *for each node* which can then be interrogated *post-hoc* (Figure 2). In this analysis, we incorporated the jumping-knowledge mechanism into an attention network framework and examine cortical segmentation performance using both the LSTM and the concatenation functions.

Given a sequence of samples *x*_1_, *x*_2_, …*x*_*t*_, an LSTM layer maintains a memory of previously observed samples in the sequence in order to learn dependencies between elements. Here, the “sequence” consists of the embeddings learned at each consecutive hidden layer, *h*^1^, *h*^2^…*h*^*L*^, representing increasingly-abstract representations of functional connectivity. We hypothesized that, because the jumping knowledge networks learn optimized node-specific network depths, these networks would be able to more-accurately segment the cortex of new data.

## 3. Data

The data used in this study come from the Human Connectome Project (HCP) (Glasser et al., 2013, 2016) and from the Midnight Scan Club (MSC) (Gordon et al., 2017). We were specifically interested in examining how models trained on one dataset would perform on another dataset. Specifically, we trained models on data from the HCP (Glasser et al., 2013), one of the highest quality MRI datasets to date in terms of spatial and temporal sampling of brain signals. We then tested our models on images from both the HCP and MSC datasets.

### 3.1. HCP Dataset

The HCP consortium collected data on a set of 1,200 young adult subjects 21–35 years of age. We utilized a subset of 268 of these datasets (22–35 years; 153 female) from the S500 data release. The HCP acquired high-resolution 0.7 mm isotropic T1w (TI = 1,000 ms, TR = 2,400 ms, TE = 2.14 ms, FA = 8°, FOV = 224 mm, matrix = 320, 256 saggital slices) and T2w images (TR = 3,200 ms, TE = 565 ms, FOV = 224 mm, matrix = 320). T1w and T2w data were pre-processed using a custom pipeline developed by the HCP (Glasser et al., 2013) using FreeSurfer (Fischl et al., 2004) to generate highly refined cortical surface meshes at the white/gray and pial/CSF interfaces. The surface meshes were spatially normalized to Montreal Neurological Institute (MNI) space and resampled to have 32k vertices. The pipeline also generated four surface-based scalar maps: cortical thickness, Gaussian curvature along the cortical manifold, sulcal depth of the cortical gyri and sulci, and a myelin density map characterizing the spatially-varying myelin content of the gray matter (Glasser and van Essen, 2011).

For each subject, the HCP acquired four resting-state functional MRI (rs-fMRI) images: TR = 0.720 s, TE = 33 ms, multi-band factor = 8, FA = 52°, FOV = 208 × 180 mm, Matrix = 104 × 90 × 72, voxel size: 2 × 2 × 2 mm. The authors refer to these four acquisitions as: REST1_LR, REST1_RL, REST2_LR, REST2_RL. The images were acquired over two separate days, such that REST1_LR / REST1_RL were acquired on 1 day, and REST2_LR / REST2_RL were acquired on another. Each session acquired 1,200 time-points, such that each BOLD session was roughly 15-min in length. These images were pre-processed using a custom pipeline developed by the HCP (Glasser et al., 2013). BOLD images were denoised using subject-ICA (Beckmann et al., 2005) and FIX (Salimi-Khorshidi et al., 2014) to automatically identify and remove spurious noise components, and motion parameters were regressed out. No additional global signal regression, tissue regression, temporal filtering, or motion scrubbing were performed. Denoised voxel time series were interpolated onto the fsaverage_LR32k surface mesh using a barycentric averaging algorithm, and then smoothed at FWHM = 2 mm to avoid the mixing of signals across gyri. Surface-mapped BOLD signals were brought into register across subjects using a multi-modal surface matching algorithm (Robinson et al., 2014) to the fsaverage_LR32 space and vectorized to CIFTI format, mapping each surface vertex to an index in a vector (toward the end of this work, we learned that different HCP data releases were processed using different versions of this surface registration algorithm; we discuss this in more depth in section 5.5). CIFTI vector indices, referred to as “grayordinates” by the HCP, are in spatial correspondence across subjects (i.e., index *i* in subjects *s* and *t* correspond to roughly the same anatomical location), such that each subject shares the same mesh topology and adjacency structure. Time-series for each session were demeaned and temporally concatenated.

The HCP consortium developed a pipeline to generate high-resolution multi-modal cortical parcellations (MMP) with 180 cortical areas using a spatial derivative based algorithm (Glasser et al., 2016) computed from resting and task-based fMRI signals, cortical thickness, myelin content, and cortical curvature. Manual editing was performed on the group-average gradient-based parcellation to ensure that boundaries conformed across feature types. Using a set of 210 independent subjects as training data, the authors trained a 3-layer neural network model to learn these boundary-based regions. The authors trained 180 classifiers, one for each cortical area, to distinguish a single cortical area from its immediately adjacent neighborhood (using a 30 mm radius neighborhood size) in a binary classification setting. At test time, the authors compared the probabilities of the predicted areal class across all classifiers in a single find-the-biggest operation. Label predictions were regularized to minimize spurious predictions and “holes” in the final parcellation. Apart from the 30 mm radius around each group-level area, the classifiers did not incorporate any spatial information at training or test time. Predictions generated from subjects in the training set were used to compute a group-average multi-modal parcellation which can be freely downloaded here: https://balsa.wustl.edu/DLabel/show/nn6K. The individual parcellations and the classifier itself have not yet been publically released.

We utilized the subject-level cortical parcellations generated by the HCP as the training set for our models. Subject-level parcellations for a subset of 449 subjects were made available by an HCP investigator (see Acknowledgements).

### 3.2. Midnight Scan Club Dataset

The Midnight Scan Club dataset consists of MRI data acquired on ten individual subjects (5 female) ranging in age from 24 to 34 years of age: https://openneuro.org/datasets/ds000224/versions/1.0.3 (Gordon et al., 2017). The MCP study acquired 5 h of resting-state data on each participant in ten 30-min acquisitions, with the goal being to develop high-precision, individual-specific functional connectomes to yield deeper insight into the reproducibility and inter-subject differences in functional connectivity.

The MSC dataset preprocessing followed a roughly similar pipeline to that of the HCP dataset. Four 0.8 mm isotropic T1w images (TI = 1,000 ms, TR = 2,400 ms, TE = 3.74 ms, FA = 8°, matrix = 224, saggital) and four 0.8 mm isotropic T2w images (TR = 3,200 ms, TE = 479 ms, matrix = 224 slices, saggital) were acquired. T1w images were processed using FreeSurfer to generate refined cortical mesh representations of the white/gray and pial/CSF tissue interfaces, which were subsequently warped to the fsaverage_LR brain surface using the FreeSurfer shape-based spherical registration method, and resampled to 164K and 32k vertex resolutions. The authors performed myelin mapping by computing the volumetric T1/T2 ratio and interpolating the voxel-wise myelin densities onto the 32k surface mesh.

MSC resting-state data were acquired using gradient-echo EPI sequences with the following parameters: TR = 2.2 s, TE = 27 ms, FA = 90°, voxel size = 4 × 4 × 4 mm. The MSC applied slice timing correction, and distortion correction using subject-specific mean field maps. Images were demeaned and detrended, and global, ventricular, and white matter signals were regressed out. Images were interpolated using least squares spectral estimation and band-pass filtered (0.009 Hz < f < 0.08 Hz), and then scrubbed of high-motion volumes. Denoised volumetric resting-state data were then interpolated onto the midthickness 32k vertex mesh. The MSC study did not perform subject-ICA and FIX to remove spurious noise components from the temporal signals.

## 4. Methods

Here, we describe processing steps applied to the HCP and MSC fMRI datasets for this analysis. We begin with the minimally pre-processed BOLD and scalar data interpolated onto the 32k surface mesh.

### 4.1. Regional Functional Connectivity

As mentioned above in sections 3.1 and 3.2, the MSC and HCP studies aligned cortical surfaces to the fsaverage_LR surface space. The result is such that, given two meshes *S* and *T*, the anatomical location of grayordinate *i* in mesh *S* corresponds to generally the same anatomical location as grayordinate *i* in mesh *T*, allowing for direct comparisons between the same grayordinates across individual surfaces.

In cases where spatial normalization of surfaces has not been performed, it would be incorrect to assume that two grayordinate indices correspond to the same anatomical locations across subjects. In order to alleviate the requirement of explicit vertex-wise correspondence across training, validation, and testing datasets, we assume that most imaging studies will first run FreeSurfer to generate subject-specific folding-based cortical parcellations (Desikan et al., 2006; Destrieux et al., 2010). We can then aggregate the high-dimensional vertex-wise connectivity features over one of these cortical atlases, as in Eschenburg et al. (2018), and simultaneously reduce the feature vector dimension. This guarantees that column indices of feature vectors represent anatomically comparable variables across individuals corresponding to connectivity to whole cortical areas rather than explicit vertex-vertex connections. These low-dimensional vectors are agnostic to the original mesh resolution and degree of spatial normalization. As long as resting-state data are collected for a given study, and that good spatial correspondence between the T1w and BOLD image can be achieved, we can apply our processing steps to this data.

Given a BOLD time series matrix *T* ∈ ℝ^32*k*×*t*^ and cortical atlas with *k* regions, we consider the set of vertices assigned to region *k* and compute the mean time-series of region *k* as:


(4) $$\begin{array}{l} {\hat{T}}_{k,t}=\frac{1}{\left|k\right|}\underset{i\in k}{\sum }T_{i,t} \end{array}$$

where $\hat{T}\in {\mathbb{R}}^{K\times t}$ is the matrix of mean regional time-series. We compute *R* ∈ ℝ^32*k*×*K*^, the Pearson cross-correlation between *T* and $\hat{T}$, where *R*_*i,k*_ represents the temporal correlation between a vertex *i* and cortical region *k*. These cross-correlation vectors are used as features to train our models.

In this analysis, we generated connectivity features using the Destrieux atlas (Destrieux et al., 2010) with 75 regions per hemisphere, as it is computed by FreeSurfer and represents a reasonably high-resolution partition of the cortical surface that we hypothesize captures vertex-to-vertex functional variability well. In section 5.5, we show how classification performance depends on which cortical atlas we regionalize over, and on which representation of functional connectivity models are trained on.

We also examined segmentation performance when models were trained on continuous representations of functional connectivity, computed by group-ICA and dual regression. As part of their preprocessing, the HCP applied group-ICA to a set of 1,003 subjects using MELODIC's Incremental Group PCA (MIGP) algorithm to compute group-ICA components of dimensions 15, 25, 50, and 100 (Smith et al., 2014). We dual-regressed these group-level components onto each subject's resting-state data to generate subject-level ICA components. These subject-level regression coefficients were fed into our models as alternative representations of functional connectivity.

### 4.2. Markers of Global Spatial Position

We also included measures of position in grayordinate space (global spatial position) as model features (Cucurull et al., 2018; Gopinath et al., 2019). Surface mesh Laplacian eigenvectors represent a spatial variance decomposition of the cortical mesh into orthogonal bases along the cortical manifold. We retained the first three eigenvectors corresponding to eigenvalues λ_1_, λ_2_, λ_3_. The eigenfunctions represent an intrinsic coordinate system of the surface that is invariant to rotations and translations of the surface mesh.

The eigendecomposition computes eigenvectors up to a sign flip (that is, the positive/negative direction of an eigenvector is arbitrary), and eigenvector ordering is not guaranteed to be equivalent across individuals. We chose a template subject and flipped (multiplied by −1) and reordered the eigenvectors of all remaining subjects with respect to this template subject via the Hungarian algorithm, to identify the lowest cost vector matching for every template-test pair (here, we minimized the Pearson correlation distance).

### 4.3. Incorporating a Spatial Prior

The models trained in this analysis represent multi-class classifiers. By default, each vertex considers every label (out of a total of 180 possible labels) as a viable candidate. This approach, however, does not take advantage of the fact that training and testing data are in spatial correspondence with one another. For example, if we know a vertex is likely to be assigned a label in the occipital lobe, we can restrict the set of candidate labels for this vertex to a subset of the possible 180 areas e.g., only those areas in the primary and higher-order visual areas. We restricted the label search space of a test vertex to only those labels with non-zero probabilities in the training set. If a given vertex *i* is never assigned label *k* in the training data, we set the estimated network probability of label *k* for vertex *i* to 0, such that it is never assigned label *k* in the test set. We implemented the application of the spatial prior by multiplying the network logits with a binary masking matrix *at test time* (e.g., the prior is not included in the model training phase).

Applying the spatial prior is only feasible if the test image surface mesh has been spatially normalized to the fsaverage_LR32 space. Given that many studies will be interested in performing multi-subject inference over surface-based maps, we believe this is a reasonable assumption to make. We examine classification performance when excluding and including a spatial prior.

### 4.4. Regional Homogeneity

We examined whether our models learned parcellations in which the features of each parcel were homogenous. We defined homogeneity for a given parcellation as in Gordon et al. (2016). Assume we are given a resting-state fMRI BOLD time series matrix *T* ∈ ℝ^32*k*×*t*^ and precomputed cortical parcellation with *L* cortical areas. For each parcel *l* ∈ *L* with *n*_*l*_ vertices, we computed the Pearson correlation matrix, $R_{l}\in {\mathbb{R}}^{n_{l}\times 32k}$, between the parcel BOLD signals with the BOLD signals of the entire cortex. We then applied the singular value decomposition as *R* = *USV*^*T*^, where *S* is the diagonal matrix of singular values σ_1_, σ_2_…σ_*N*_. Gordon et al. (2016) defined homogeneity as $\rho_{l}=100*(\sigma_{1}^{2}/\sum_{i=1}^{k}\sigma_{i}^{2})$, the percent of variance explained by the first principal component. The variance captured by the first component describes how well a single vector explains the functional connectivity profiles of a given cortical parcel—the larger the variance explained, the more homogeneous the parcel connectivity. We computed an estimate of functional homogeneity for each parcel and averaged the estimates across all parcels.

For scalar features (e.g., myelin density), we estimated homogeneity as the ratio of within-parcel variance to between-parcel variance. For each parcel *l* ∈ *L* and feature *F* ∈ ℝ^32*k*^, we computed the mean, μ_*l*_, and variance, $\sigma_{l}^{2}$ of the parcel-wise features. Homogeneity is estimated as $\sum_{i=1}^{L}\left(\sigma_{l}^{2}-\bar{\sigma^{2}}\right)/\sum_{i=1}^{L}{\left(\mu_{l}-\bar{\mu }\right)}^{2}$, where $\bar{\sigma^{2}}$ and $\bar{\mu }$ are the average variance and average mean estimates across all parcels. A smaller value represents more homogeneous parcels. This measure of homogeneity is a dimensionless quantity that allows for the comparison of estimates across datasets and features.

### 4.5. Model Training and Parameter Selection

We implemented each graph neural network model using the Python package Deep Graph Library (DGL) and PyTorch (Wang et al., 2020). Code developed for this analysis for training these models can be found here: https://github.com/kristianeschenburg/parcellearning/.

We split the 268 HCP subjects into 100 training samples, 20 validation samples, and 148 test samples. For parameter optimization, we trained models on three types of datasets: (1) 100 15-min images (REST1_LR session for each subject), (2) 100 60-min images (temporal concatenation of all four rfMRI sessions), and (3), 400 15-min images (four independent rfMRI sessions per subject). We used a validation dataset of 20 subjects of the same scanning duration as the training data to determine when to stop training. We examined the performance of each model on test hold-out test set of different scanning durations: 15-min (four independent rfMRI sessions), 30-min (concatenation of two 15-min rfMRI sessions acquired on the same day), and 60-min (temporal concatenation of all four 15-min rfMRI sessions). The outcome variable to be predicted was the subject-level parcellation provided to us by MG. We performed similar temporal concatenation of the MSC data, concatenating the original ten 30-min sessions into five 60-min sessions, two 150-min sessions, and one 300-min session.

The features used for parameter optimization were the regionalized functional correlations between each cortical vertex and all regions in the Destrieux atlas, the first three Laplacian eigenvector embeddings capturing global location information, and four scalar maps corresponding to sulcal depth, Gaussian curvature, myelin density, and cortical thickness for a total of 81 features at each vertex. We concatenated these features column-wise into a matrix for each subject.

We refer to the graph convolution network, graph attention network, and jumping knowledge network as “GCN,” “GAT,” and “JKGAT,” respectively. We compared the performance of these algorithm variants to a simple linear feed-forward neural network (“baseline”) where only the features at each vertex were used to classify cortical nodes (no adjacency information is incorporated into the learning process). We optimized model performance over network depth, number of hidden channels per layer, feature dropout rate, number of attention heads (GAT and JKGAT only), and aggregation function (JKGAT only). The “default” parameters are 3 layers, a dropout rate of 0.1, 32 hidden channels, 4 attention heads per layer, and an LSTM aggregation function. We varied one parameter at a time: for example, when comparing networks with 3 layers vs. 6 layers, all other parameters are fixed to the default values.

For training, we used the cross entropy loss implemented in Pytorch, a LeakyReLU activation function with a negative slope of 0.2, and Adam optimization with a weight decay rate of 0.0005 and L2 weight regularization of 0.005. We trained in mini-batches of size *s* = 10 graphs and accumulated the gradients for each batch before computing the gradient update. We trained for 1,000 epochs using an early stopping criteria evaluated on the validation loss. At each iteration, we retained the model if the current validation loss was lower than the previous validation loss. If validation loss did not decrease for 150 epochs, training was terminated and the best performing model was saved. In practice, we found that few of the models trained for more than 1,000 epochs.

## 5. Results

We first examine the best performing model of those we considered in our analysis, and discuss the classification accuracy and reproducibility of parcellations predicted by this model in relation to parcellations computed by Glasser et al. (2016), which we call “ground truth” in what follows. We define classification accuracy as the percentage of correctly predicted vertex labels relative to the ground truth maps. We then show broadly how algorithm choice, network architecture, and training and testing image scan duration affect overall model performance. Finally, we illustrate how classification performance is related to the features used during model training and testing.

### 5.1. Prediction Accuracy in the Best Performing Model

Network optimization was performed using labels provided by Matthew Glasser (see section Acknowledgments) using subject data from the S500 HCP release. As mentioned in section 3.1, the S1200 data release uses a different surface registration algorithm, producing subject-level resting-state data that is better aligned with the labels provided by Glasser. Final model evaluation was performed using this S1200 data. The best performing model was the 6-layer graph attention network (GAT), with 4 attention heads per layer, 32 hidden channels per layer, and a dropout rate of 0.1, and incorporated a spatial prior at test time. When trained on features computed using ICA, this model achieved a mean classification accuracy of 79.91% on the S1200 subjects. We henceforth refer to this model as the “optimal” model, and discuss results associated with this model below.

**Figure 3:**
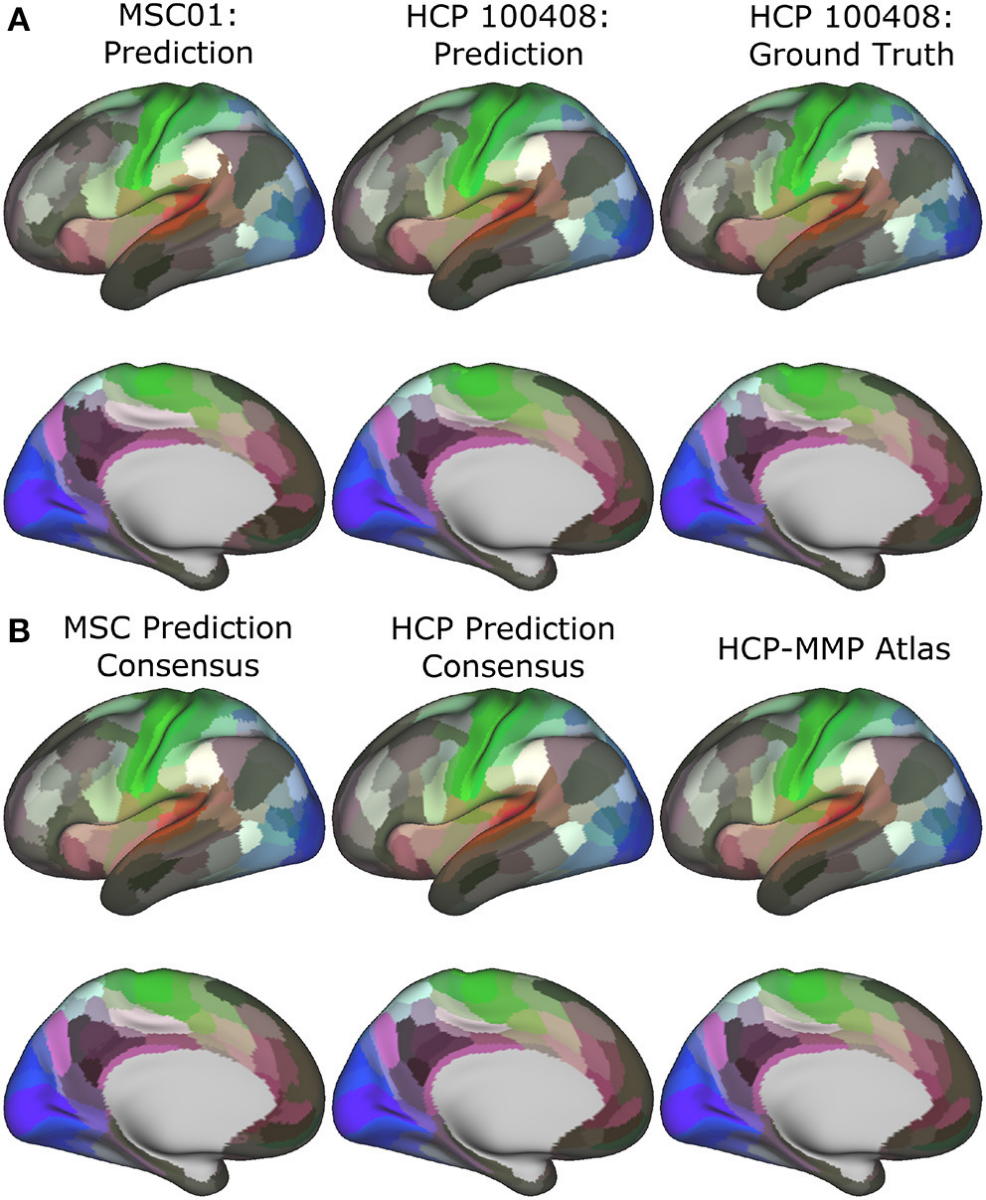
Subject-level **(A)** and group-level **(B)** predictions generated by the optimal model in the MSC (left) and HCP (middle) datasets.

In Figure 3A, we show predicted parcellations computed using this model for exemplar HCP and MSC test subjects. Predicted subject-level parcellations closely resemble the “ground truth” maps generated by Glasser et al. (2016) (see Supplementary Material for additional examples of predictions generated by each model). No specific contiguity constraint was imposed on the parcellations; it is inherent in the graph neural network models. Subjects from the MSC dataset do not have corresponding ground truth maps against which to compare their predictions. In Figure 3B, we show consensus predictions for each dataset, compared against the publicly released HCP-MMP atlas. Consensus predictions were computed by assigning a vertex to the label most frequently assigned to that vertex across the individual test subject predictions. We see that both consensus predictions closely resemble the HCP-MMP atlas—however, the consensus map derived from the MSC subjects shows noisy parcel boundaries and disconnected areal components (lateral and medial prefrontal areas).

**Figure 4:**
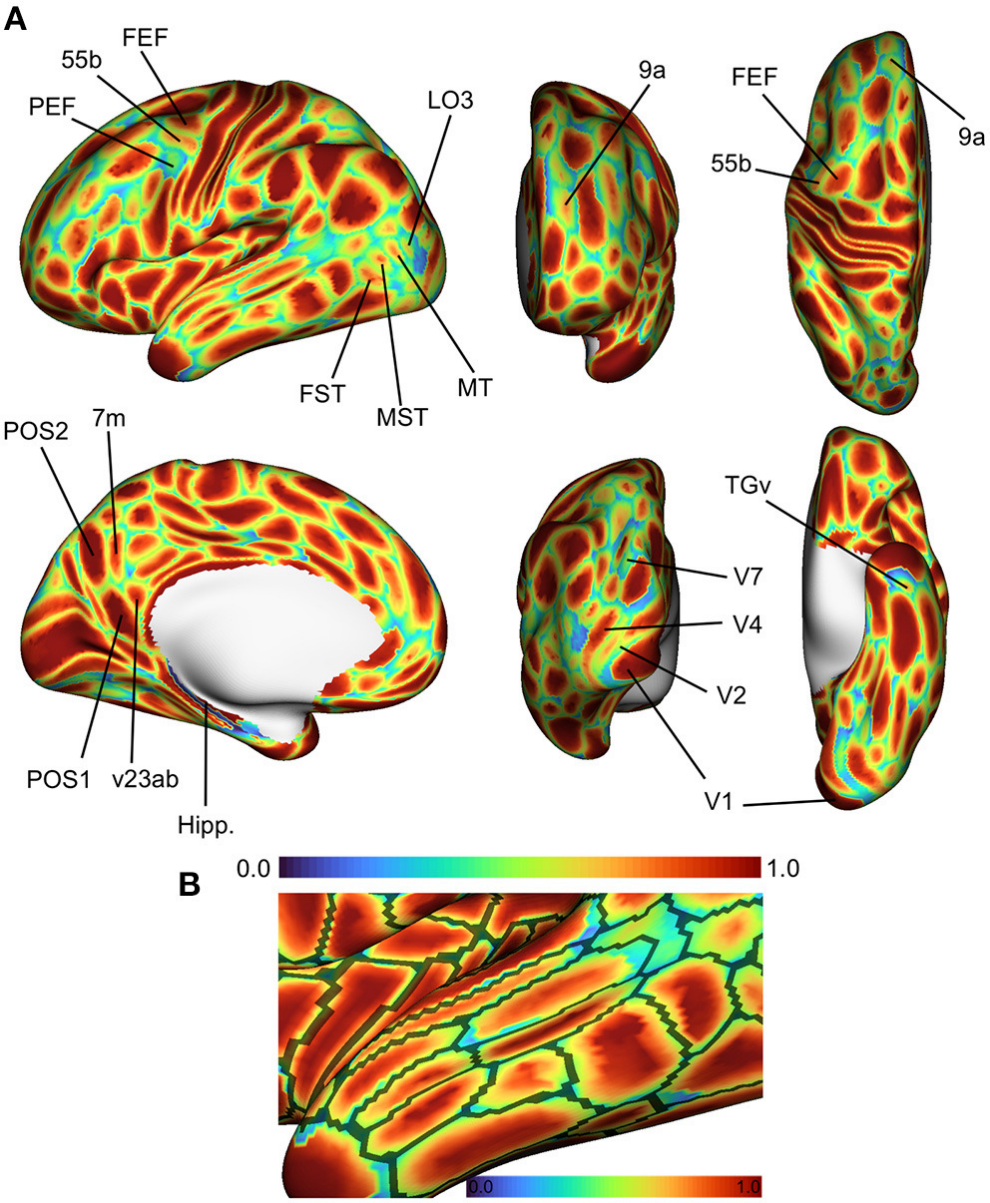
Average accuracy maps for the HCP test set using the optimal model, computed by averaging the classification error maps across all HCP dataset test subjects. **(A)** Blue (0.0) = vertex incorrectly classified in all test subjects; Red (1.0) = vertex correctly classified in all test subjects. Areas in the lateral prefrontal and ventral/dorsal occipital areas showed the highest error rates. **(B)** Errors occur most frequently at the boundaries of cortical regions. Black lines represent areal boundaries of the consensus prediction parcellation.

Figure 4 shows the spatial distribution of classification accuracy rates averaged across all subjects in the HCP test set. Average accuracy is shown as a map distributed over the cortex, with values ranging between 0 (blue; vertex incorrectly classified in all subjects) and 1 (red; vertex classified correctly in all subjects). Vertices near the centers of cortical regions were classified correctly more frequently, while prediction errors tended to be distributed near the boundaries of cortical regions. To some degree, this effect can be attributed to the idea that boundaries between putative cortical areas represent segments of the cortex with changing biological properties. In developing a statistical model to assign a vertex to one cortical area or another, vertices at region boundaries will have more ambiguous label assignments simply due to the fact that their feature vectors are sampled from a space with greater distributional overlap across various cortical areas. However, another explanation is that MRI resolution is low with respect to cortical functional features like cell columns. Consequently, this means that voxel-wise measurements reflect mixtures of connectivity patterns due to partial volume effects, thereby reducing the ability of a statistical model to distinguish between two cortical areas at parcel boundaries.

While errors globally tended to be concentrated at region boundaries, some cortical areas showed higher error rates than others. Of note are higher error rates for cortical areas in the superior temporal areas in the fundus and medial superior temporal regions (FST, MST, MT, and V4t), and lateral higher-order visual areas (LO1, LO2, LO3). In the lateral prefrontal area, we found that the premotor eye field (PEF) shows higher error rates relative to adjacent regions (55b and frontal eye field, FEF). Glasser et al. (2016) identified three unique topologies (typical, shifted, and split) for area 55b that varied across subjects, which might to some degree explain the higher error rates in area PEF.

We quantified the relationship between the spatial distribution of errors and their distance to cortical areal boundaries. We computed the fraction of misclassified vertices that occurred at a geodesic distance of *k* edges (geodesic hops) from any cortical areal boundary. Using the default model parameters and regionalized features, we examined this distribution of errors as function of distance (Supplementary Material). Over 50% of misclassified vertices occurred at the region boundaries i.e., those vertices in the ground-truth parcellations that are directly adjacent to different regions, and roughly 30 and 12% of misclassified vertices were 1 and 2 edges away from areal boundaries, respectively. The simple feed-forward network misclassified vertices further away from region boundaries, while the three graph neural networks tended to misclassify only vertices close to the boundary.

Although the MSC subjects do not have corresponding ground truth maps, the data is in spatial correspondence with the fsaverage_LR32 map. We computed the correspondence of maps predicted on the MSC subjects with the HCP-MMP atlas in order to gain insight into the accuracy of these predictions. Mean correspondence of predictions computed on the MSC and HCP datasets with the HCP-MMP atlas was 70.04 and 84.35%, respectively (Supplementary Material).

**Figure 5:**
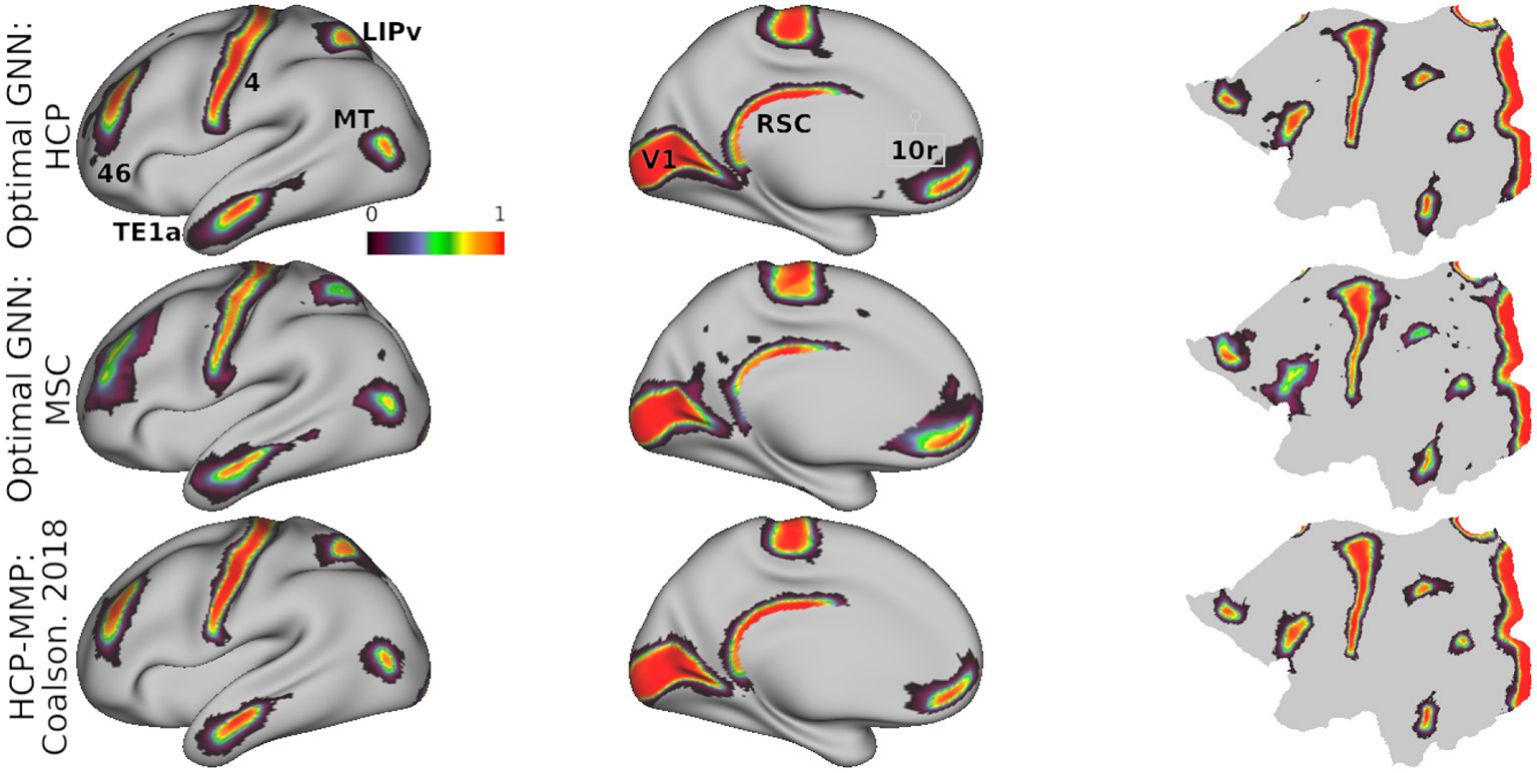
Mean model probabilities for a subset of cortical areas for the HCP **(top)** and MSC **(middle)** datasets computed using the optimal model, and the MMP binary class probabilities from Glasser et al. (2016) and Coalson et al. (2018) **(bottom)**. Probabilistic maps are illustrated for areas V1, 46, TE1a, LIPv, MT, RSC, and 10r. These maps are thresholded at a minimum probability value of 0.005, the probability of randomly assigning a vertex to one of the 180 cortical areas.

Mean model probabilities computed by the optimal model for a set of cortical areas are illustrated in Figure 5, showing that areal probabilities are local in nature and restricted to precise anatomical locations. Individual areal probabilities computed by Glasser et al. (2016) and Coalson et al. (2018) using their binary classifier are shown in the bottom row. Probability estimates in the HCP dataset mirror those estimated by the original HCP classifier (Glasser et al., 2016), indicating that our model faithfully learns the proper spatial extent of each cortical areal. Estimates in the MSC dataset were slightly more diffuse and less confident (see areas V1 and 46), such that probability mass was assigned to more disparate areas of the cortex, relative to probabilities estimated in the HCP dataset.

### 5.2. Model Predictions Are Reproducible Across Scanning Sessions

The HCP acquired four 15-min resting-state acquisitions per subject, while the MSC acquired ten 30-min resting-state acquisitions per subject. We examined how reliable predictions generated from each resting-state session were within subjects, and how this reliability related to the scanning duration. For a given subject, we estimated session-specific reproducibility using datasets of the same scan duration. We defined reproducibility using the Dice coefficient, which measures the similarity of two images. The Dice coefficient between sets *J* and *K* is defined as


(5) $$\begin{array}{l} Dice\left(J,K\right)=\frac{2*|J\cap K|}{\left|J|+|K\right|} \end{array}$$

**Figure 6:**
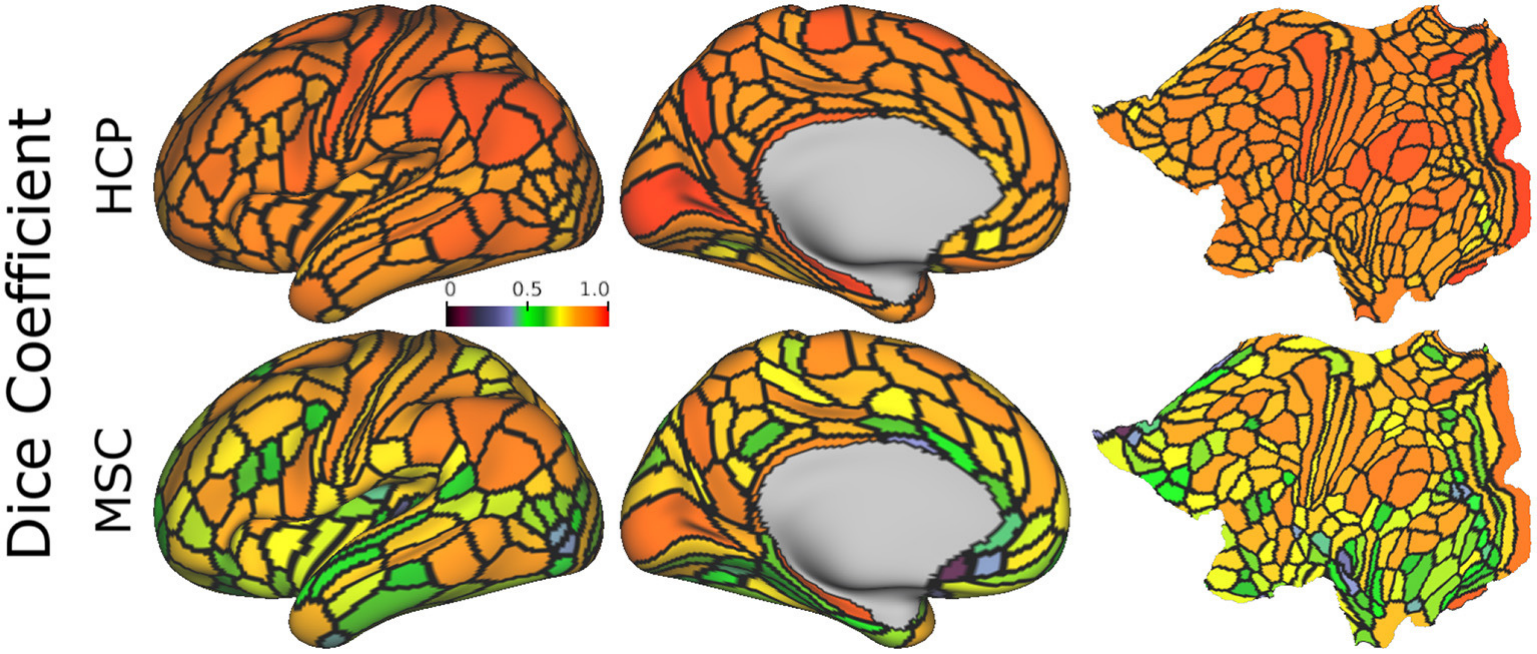
Mean areal Dice coefficient estimates, computed using the optimal model on 15-min HCP data (4 repeated sessions) and 30-min MSC data (10 repeated sessions), normalized with the same color map. Estimates are computed for each area, and averaged across all subjects.

Figure 6 shows the mean areal Dice coefficients for each dataset from predictions computed using the optimal model. Predictions made on the HCP dataset were more reproducible across the entire cortex than predictions on the MSC dataset. In both datasets, sensory/motor and areas near the angular and supramarginal gyri were most reproducible. The visual cortex showed high reproducibility in area V1, while areas V2-V4 were less reproducible.

**Figure 7:**
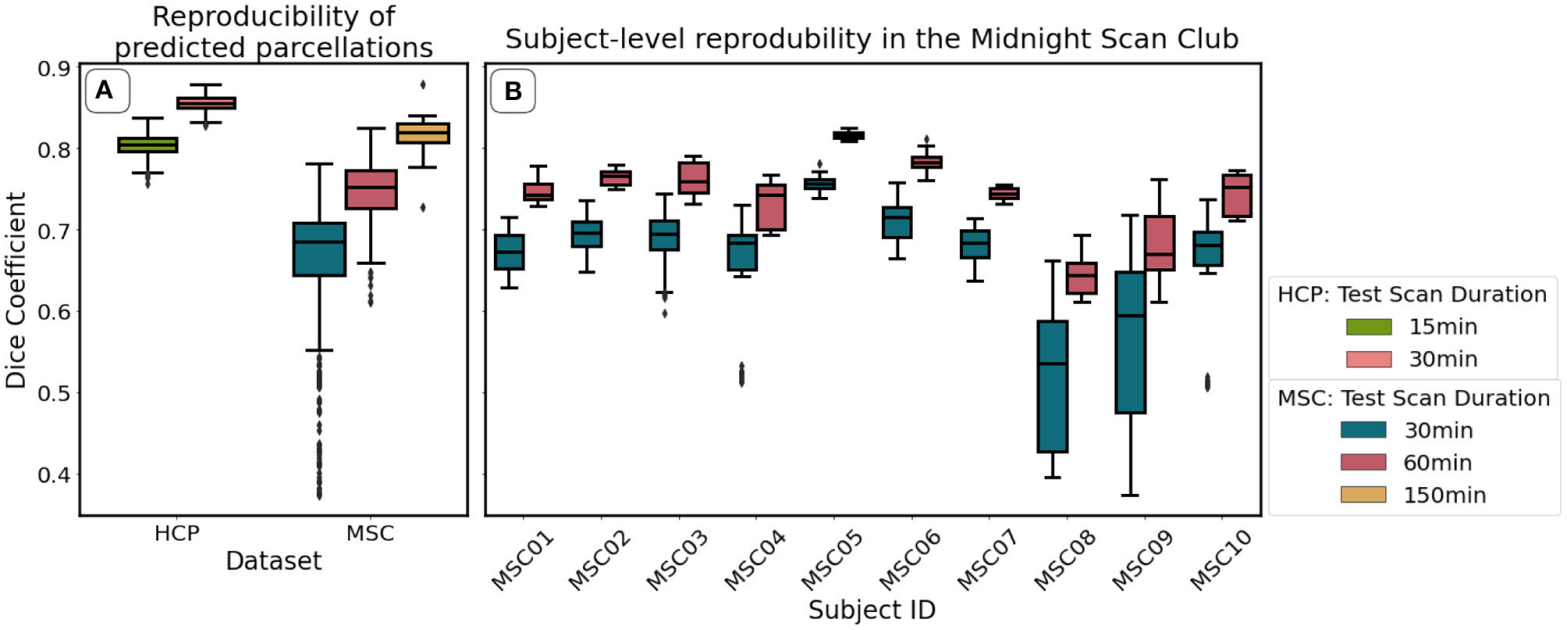
Reproducibility of predicted maps generated by the optimal model, as measured using the Dice coefficient. We show mean reproducibility estimates for each dataset **(A)**, and subject-level estimates in the Midnight Scan Club **(B)**. Estimates for 60 min (HCP) and 300 min (MSC) durations are not shown in **(A)** because there is only one image per subject for these durations. Similarly, estimates for 150 min durations are not shown in **(B)** because there is only a single scalar estimate per subject.

Figure 7A, shows mean reproducibility estimates computed on the HCP and MSC datasets. Predictions for both datasets were highly reproducible across repeated scanning sessions, and reproducibility increased with increasing scan duration. Mean Dice coefficient estimates in the HCP dataset were 0.81 and 0.86 for the 15- and 30-min durations. In the MSC dataset, the mean Dice coefficients were 0.69, 0.76, and 0.82 for the 30-, 60-, and 150-min durations. When fixing scan duration (e.g., 30-min durations), HCP data were more reproducible than the MSC data. One feature that we could not evaluate directly was the reproducibility of the ground truth maps. Glasser et al. (2016) reported maximum and median Dice coefficient estimates of 0.75 and 0.72 for repeated scans on HCP participants, indicating that our classifier learned parcellations that were more reproducible than those generated by the binary classifier.

Figure 7B illustrates subject-level reproducibility estimates in the MSC dataset. Predictions for subject MSC08 were significantly less reliable, relative to the other subjects. Gordon et al. (2017) also identified MSC08 as having low reproducibility with respect to various graph theoretical metrics computed from the functional connectivity matrices. They noted that subject MSC08 reported restlessness, displayed considerable head motion, and repeatedly fell asleep during the scanning sessions.

Area-level topologies were also reproducible across scanning sessions (Supplementary Material). Glasser et al. (2016) identified three unique topologies of area 55b, corresponding to a “typical,” “shifted,” or “split” organization pattern, relative to the group-average cortical map. We were able to identify these same unique topologies in individual subjects, indicating that graph neural networks are identifying the unique connectivity fingerprints of each cortical area, and not simply learning where the parcel is. When we examined the predictions generated by the optimal model on the four independent 15-min scanning sessions, we found that, within a given subject, the topological organization of area 55b was reproducible. Allowing for some variability in prediction boundaries and location due to resampling of the connectivity data and partial volume effects, this indicates that the graph neural networks are learning subject-specific topological layouts that incorporate their unique connectivity and histology patterns.

### 5.3. Parcellations Learned by GNNs Are Homogeneous in Their Scalar and Connectivity Measures

If a model is in fact learning unique, discrete areas, the distribution of biological features in these areas should be relatively homogeneous. Unsupervised learning clustering algorithms designed to parcellate the cortex often incorporate objective functions that attempt to maximize within-parcel similarity and minimize between-parcel similarity. On the other hand, gradient-based approaches, like those proposed in Gordon et al. (2016), Wig et al. (2014), and Schaefer et al. (2018), do not directly maximize an objective function in this manner, but rather identify putative areal boundaries by identifying where biological properties change dramatically in a small local neighborhood. It is assumed that this biological gradient captures differences in homogeneity between adjacent cortical areas. In order to group cortical voxels together, these voxels must inherently share some physical or biological traits.

We computed homogeneity estimates as described in section 4.4. In order to compare the homogeneity and variance estimates between predicted parcellations, we fixed the features used to compute these estimates. For a given subject, we computed functional homogeneity using that subject's 60-min BOLD signal (HCP), or the 300-min BOLD signal (MSC). In this way, the only variable that changed with respect to the homogeneity estimate is the cortical map itself. We could then make meaningful quantitative comparisons between estimates for different maps, with respect to a given dataset.

**Figure 8:**
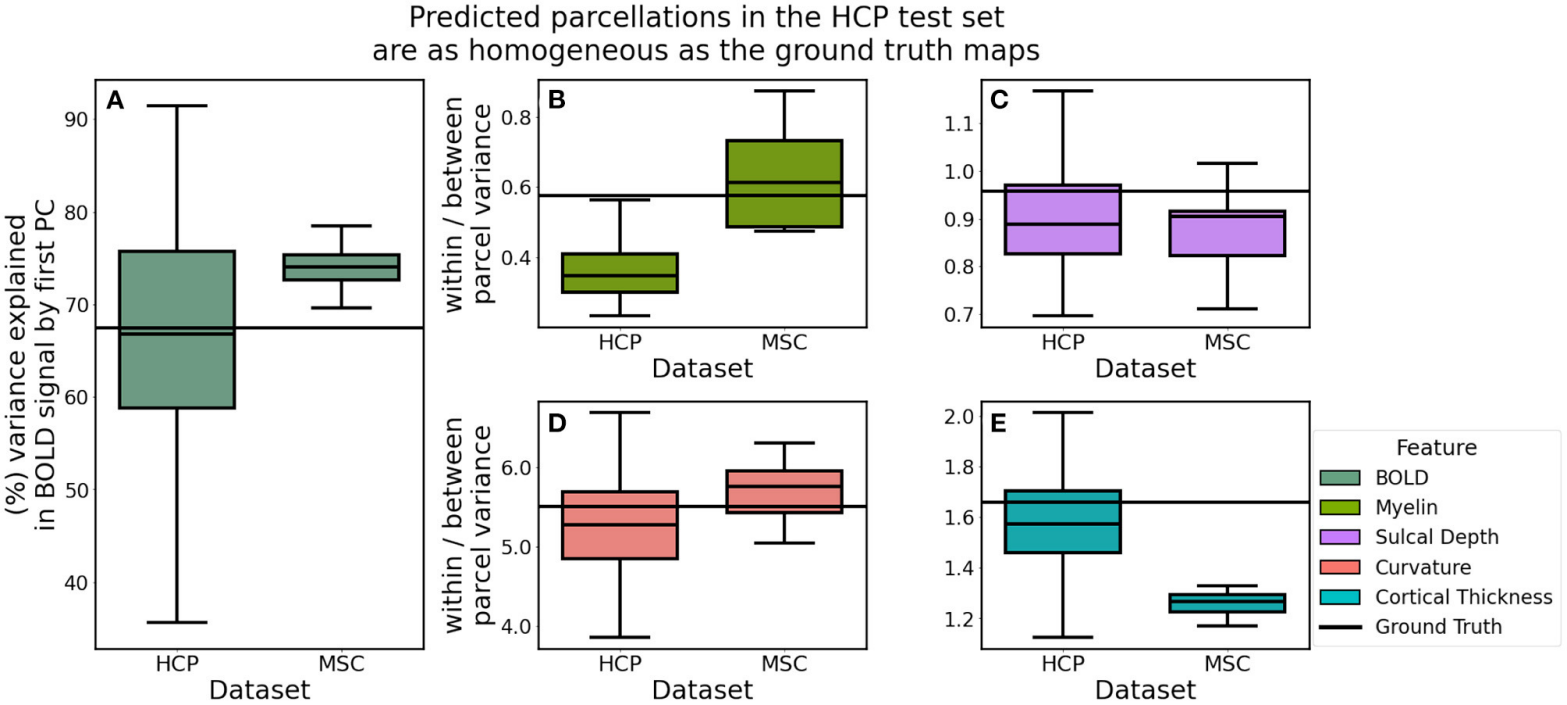
Homogeneity of predicted parcellations in the HCP and MSC datasets using the optimal model. **(A)** Predicted parcels in the HCP test set explained as much variability in the functional connectivity as the ground truth parcels. **(B–E)** Predictions in the MSC had more variable myelin content and less variable cortical thickness estimates, relative to the HCP predictions.

Cortical maps predicted in the HCP dataset explained, on average, 67.03% of the functional variation while MSC predictions explained 72.90% (*t*: −3.137, *p*: 0.007) (Figure 8). We hypothesized that parcellations predicted in the HCP dataset would be more homogeneous, relative to those learned in the MSC dataset, due to the fact that the MSC imaging data were acquired with lower spatial resolution than that acquired by the HCP and therefore subject to greater partial volume effects. Homogeneity of myelin (*t*: −0.910, *p*: 0.377) and sulcal depth (*t*: 1.043, *p*: 0.320) was not statistically different between the two datasets, while curvature was less variable in the HCP dataset (*t*: −2.423, *p*: 0.029). Contrary to our hypothesis, cortical thickness was less variable in the MSC dataset (*t*: 11.562, *p*: 0.000). This is likely a consequence of using a dimensionless representation of homogeneity, which is internally normalized for each dataset as a ratio of the within-to-between parcel variances. This metric allows for the direct comparison of homogeneity estimates across datasets, instead of representing the raw variance estimates.

We compared homogeneity estimates in the predicated HCP parcellations to estimates computed for the ground truth maps using paired *t*-tests. Predicted and ground truth maps both explained roughly 67% of the functional variation (*t*: −0.305, *p*: 0.761). Myelin (*t*: 0.176, *p*: 0.860) and curvature: (*t*: −1.746, *p*: 0.083) variation were not statistically different between the two groups. However, predictions were more homogeneous than the ground truth maps with respect to sulcal depth (*t*: −4.442, *p*: 0.000) and cortical thickness: (*t*: −2.553, *p*: 0.012).

### 5.4. Network Architecture Impacts Model Performance

As noted in section 5, we first optimized over network algorithms and architectures using the S500 dataset, and then utilized the S1200 dataset for model evaluation. We fixed the features used for network optimization to the regionalized connectivity features. We examined how varying each network parameter impacted model classification accuracy (Table 1). As mentioned in section 5.1, the best performing model was the GAT network with 6 layers with a classification accuracy of 67.60% on the S500 dataset (significantly inferior to the performance of the same network on S1200 data, with an accuracy of 79.91%). We found that optimal performance for the GAT and GCN networks was achieved with 6 layers, 9 layers for the JKGAT, and 3 layers for the baseline model. In general, classification accuracy increased with the number of attention heads, and number of hidden channels, while classification accuracy decreased with increasing feature dropout rates. Using an LSTM aggregation function rather than a simple concatenation marginally decreased classification accuracy for the jumping-knowledge networks. In contrast to our predictions, we found that the GAT networks slightly outperformed the more flexible JKGAT networks for most parameterizations.

**Table 1:** Model classification accuracy as a function of network architecture and parameterization.

		**Model**			
**Parameter**	**Value**	**Baseline (%)**	**GCN (%)**	**GAT (%)**	**JKGAT (%)**
Network depth	3	62.64	64.93	67.02	66.71
	6	61.13	65.14	**67.60**	67.33
	9	57.72	64.76	67.36	67.42
Hidden channels	16	60.54	62.60	66.37	66.12
	32	62.64	64.93	67.02	66.71
	64	63.84	66.24	67.15	67.15
Dropout rate	0.1	62.64	64.93	67.02	66.71
	0.3	60.74	63.94	66.72	66.58
	0.5	58.34	63.10	65.45	65.39
	0.7	55.63	61.18	62.70	62.60
Attention heads	4			67.02	66.71
	8			67.39	67.30
	12			67.56	67.29
Aggregation function	concat				66.85
	lstm				66.71

*Models were trained on 400 15-min datasets, and tested on 60-min test data using the S500 dataset. Boxed values indicate the default parameter values. The best performing model was the GAT network with 6 layers, achieving a mean classification accuracy of 67.60%. Values in bold are the mean classification accuracy of the best model, trained on resting-state connectivity features computed by regionalizing time-series over the Destrieux cortical atlas (see Section 4.1)*.

We used a fixed validation dataset of 20 subjects to determine when to stop model training and evaluated the performance of our models using a fixed test dataset of 148 subjects. In order to determine the reliability of our accuracy estimates, we computed the standard error of classification accuracy for each model using a bootstrapped approach (Supplementary Material). We randomly sampled 100 test subjects, with replacement, out of the 148, and computed the mean accuracy for each sample, for each model. We repeated this process 1,000 times, and computed the variability of these bootstrapped estimates. Standard error estimates were less than 0.5%, indicating that test set accuracy estimates are robust with respect to resampling of the test dataset.

We examined how classification accuracy in the HCP dataset was related to the scanning duration of training and testing datasets using the default model parameters (as defined in section 5). When fixing test scan duration, classification accuracy improved as the training dataset size increased for all model types, with maximum accuracy achieved by graph attention network models trained on 400 15-min duration datasets (Supplementary Material). When training dataset size and training scan duration were fixed, longer test image duration yielded more accurate predictions across the board. Predictions on 60-min test data were more accurate than those computed on 30-min images, which in turn were more accurate than those generated from 15-min images (Supplementary Material). However, models trained on 15-min data performed best when tested on 15-min data, and models trained on 60-min data performed best when tested on 60-min data (Supplementary Material) indicating an interaction between training and testing scan duration. Similarly, when fixing training and testing scan duration, we found that including the spatial prior significantly improved classification accuracy in all architectures.

### 5.5. Incorporating Functional Connectivity Improves Model Performance Beyond Spatial Location and Scalar Metrics

After identifying the optimal network architecture, we examined how model performance varied as a function of which features the model was trained on. Briefly, we delineated three broad feature types: (1) scalar features corresponding to myelin, cortical thickness, sulcal depth, and cortical curvature (2) global location features corresponding to the spectral coordinates computed from the graph Laplacian and (3) connectivity features computed from the resting-state signal. In our primary analysis, we utilized connectivity features computed by regionalizing over the Destrieux atlas (75 folding-based cortical areas). We compared these features against those computed using the Desikan-Killiany atlas (35 folding-based cortical areas) and the Yeo-17 resting-state network atlas (Yeo et al., 2011). The Yeo-17 atlas is a functional atlas of discretized resting-state networks, computed via independent component analysis. We identified the connected components of each of the 17 resting-state networks and excluded component regions with sizes smaller than 10 vertices, resulting in a map of 55 discrete functionally-derived subregions of the cortex. We also examined the performance of models trained on continuous, overlapping connectivity features representing resting-state networks computed using group-ICA and dual regression.

**Figure 9:**
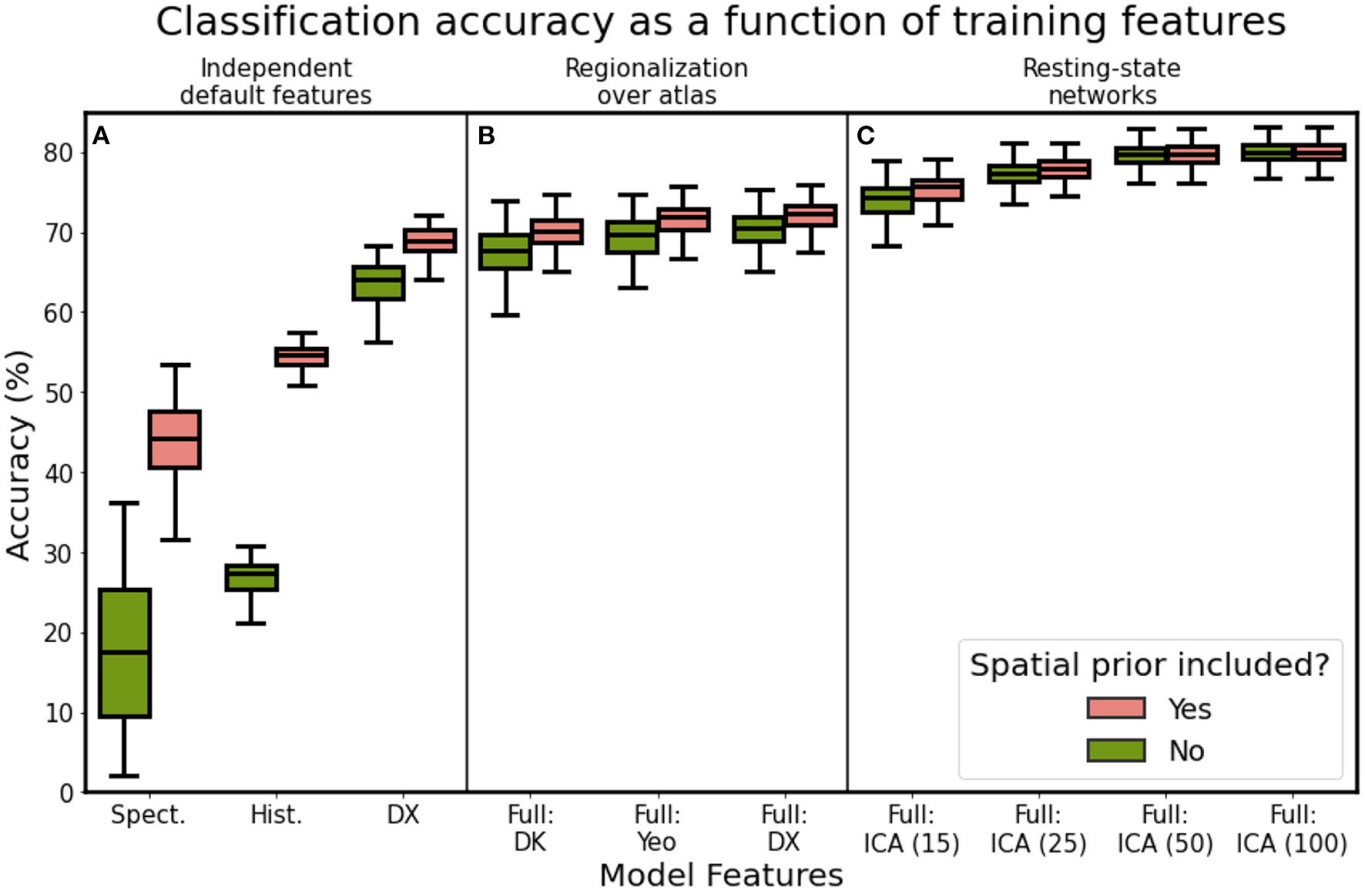
Classification accuracy as a function of model features, using the optimal model architecture for **(A)** single feature types, **(B)** regionalization over different cortical atlases, and **(C)** independent component analysis features. Refer to Table 2 for a description of each feature set.

Computing connectivity features over the Destrieux atlas yielded increased classification accuracy over the Desikan-Killiany atlas (72.01 vs. 70.08%; paired *t*: 25.197, *p*: 0.000; see models “Full-DX” and “Full-DK”). We hypothesized that computing connectivity features over a functionally-aware parcellation (Yeo-17) would yield a significant improvement in classification accuracy, relative to the Destrieux atlas, but this was not the case (see “Full-DX” vs. “Full-YEO” in Figure 9). Models trained on the Yeo-17 features had a mean classification accuracy of 71.58% (paired *t*: 1.916, *p*: 0.057). Training on spatial location or histological features alone yielded mean classification accuracies of 44.10 and 54.45%, respectively (Figure 9A). However, training on features defined by resting-state ICA components had clear performance benefits. Models trained on ICA dimensions of 15, 25, 50, and 100 generated mean classification accuracies of 75.34, 77.79, 79.68, and 79.91%, respectively (Figure 9C). Similarly, incorporating the prior mask also improved model performance. However, the mask added diminishing returns, with the better-performing models benefiting less from its inclusion. Models trained on higher-dimensional ICA resting-state networks (50 and 100 networks), performed almost as well without the spatial prior as they did with it.

**Table 2:** Feature combinations tested by our optimal model.

**Feature sets**							
	**Full feature sets**				**Connectivity**	**Scalar**	**Location**
	**DK (F)**	**DX (F)**	**YEO (F)**	**ICA (F)**	**DX**	**Hist**.	**Spect**.
Thickness	+	+	+	+		+	
Curvature	+	+	+	+		+	
Myelin	+	+	+	+		+	
Sulcal depth	+	+	+	+		+	
Laplacian	+	+	+	+			+
Desikan (DK)	+						
Destrieux (DX)		+			+		
Yeo-17 (YEO)			+				
ICA-RSN				+			

*Features included in a model are marked by a “+.” “Full” models include histological features, global position information, and functional connectivity signals*.

**Figure 10:**
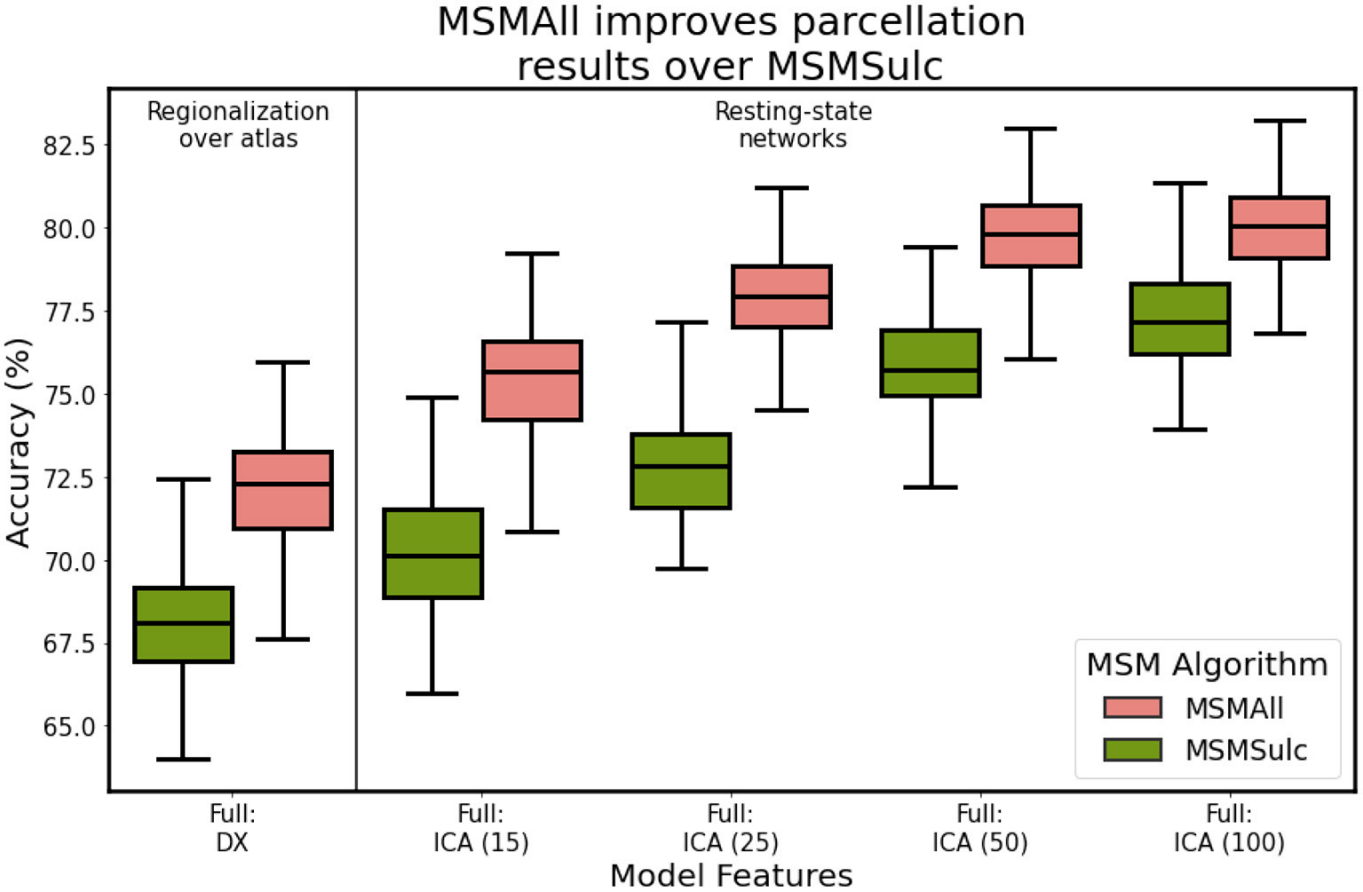
Classification accuracy as a function of HCP data release and corresponding multi-modal surface matching algorithm. S500: MSMSulc (Robinson et al., 2014), S1200: MSMAll (Robinson et al., 2018).

Late into our analysis, we learned of differences in the preprocessing steps used to generate the minimally-preprocessed HCP resting-state data, and to generate the subject- and group-level HCP-MMP parcellations. Specifically, the S500 and S1200 data releases were preprocessed using different surface registration algorithms: MSMSulc and MSMAll (Robinson et al., 2014, 2018). A consequence of these preprocessing differences is that data from the S1200 release is better aligned with the subject-level labels provided by Glasser. After performing network optimization using the S500 data, we evaluated final model performance on the S1200 dataset. Figure 10 illustrates model performance after training on each independent dataset. We found that utilizing the S1200 dataset showed significant improvements in mean classification accuracy by upwards 5%, relative to the S500 dataset. This indicates that the surface registration algorithm choice plays a critical role in cortical segmentation quality.

## 6. Discussion

In this analysis, we presented a general cortical segmentation approach that, given functional connectivity information and a set of corresponding training labels, can generate cortical parcellations for individual participants. This approach to segmenting the cortex requires accessible MRI acquisition sequences and standard morphological parcellations as inputs. We compared three different graph neural network variants to a baseline fully-connected network. We found that, in all cases, graph neural networks consistently and significantly outperformed a baseline neural network that excluded adjacency information. We identified the best performing model and explored its performance with respect to various metrics like segmentation accuracy, prediction reliability, and areal homogeneity in two independent datasets.

Predictions generated for both the HCP and MSC datasets were highly reproducible. However, we found that nearly twice as much resting-state data was required in MSC subjects to achieve the same reproducibility estimates as in the HCP data. Predictions generated on the HCP dataset were more reproducible than the ground truth maps themselves (Glasser et al., 2016), while predictions in the MSC data were roughly as reproducible as the ground-truth parcellations. This may in part be due to the way we trained our models. Models were trained on repeated samples of BOLD images, such that for a given training subject, models were shown four BOLD datasets. This likely enabled the models to better learn the mapping between a given subject's unique BOLD signature, and its cortical map. Another possible explanation is that the ground truth maps were generated using a linear perceptron model, which does not take into account any spatial relationships between data points, while graph neural networks do take this spatial structure into account. It is likely the case that the perceptron model could not adapt to utilize spatial dependencies in the BOLD signal in local neighborhoods and thereby failed to fully learn unique subject-specific connectivity fingerprints, and consequently learned more variable parcellations.

The optimal model predicted parcellations that were as homogeneous as the ground truth maps when considering multidimensional connectivity features and univariate scalar features. Though the models considered in this analysis are capable of learning parcels that capture inter-areal variation of functional brain connectivity and other cortical features, it is worth noting that homogeneity as a measure of parcellation quality is an imperfect metric and should be used judiciously. For example, the primary sensory areas can be further divided into five somatotopic subregions corresponding to the upper and lower limbs, trunk, ocular, and face areas (Glasser et al., 2013). These subdivisions correspond well with task-based fMRI activity and gradients in myelin content, indicating that the parcels learned by GNNs in our analysis still incorporate significant variability due to the aggregation of signals from different somatosensory areas. While learning homogeneous regions is important in order to effectively capture spatial biological variation, maximizing homogeneity was not the training criterion for this analysis.

As noted in section 3, the MSC study applied different preprocessing steps than the HCP. Specifically, the MSC did not perform FIX-ICA to remove noise components from the BOLD images and utilized the FreeSurfer spherical surface registration to bring surfaces into spatial correspondence with one another instead of the multi-modal surface matching algorithm (Robinson et al., 2014, 2018). Given that the MSC dataset did not have “ground truth” labels against which we could compare predictions made on the MSC data, we compared predictions against the HCP-MMP atlas (Glasser et al., 2016). As expected, predictions generated on the HCP dataset more closely resembled the HCP-MMP atlas than predictions made from the MSC dataset (the HCP-MMP atlas was derived as a group-average of individual ground truth parcellations). Nevertheless, we found that correspondence of MSC predictions with the atlas followed similar trends with respect to testing image duration. We believe some discrepancy in results between the HCP and MSC datasets can be attributed to the differences in dataset-specific preprocessing choices noted above, although the relationship between methodological choices and parcellation outcome requires future analyses. Performance differences across the two datasets are also possibly a result of the models learning characteristics inherent to the training (HCP) dataset, and thereby performing better on hold out subjects from that same dataset.

Our optimal model was the 6-layer graph attention network, trained and tested on resting-state network components computed using a 50-dimensional ICA. This model performed as well with the spatial prior as it did without. However, models trained on regionalized connectivity features benefited from including the spatial prior. We believe it would be prudent for future studies to include a spatial prior of some form into their classification frameworks. Interestingly, predictions on HCP test subjects resembled the HCP-MMP atlas more closely than they resembled their ground truth counterparts, which might in part be driven by the specific form of the prior. We made the assumption that cortical map topology is relatively conserved across individuals. This assumption may be too conservative and may reduce model sensitivity to atypical cortical connectivity patterns. Nevertheless, there is evidence our GNN models learn subject-specific topologies of cortical areas, rather than simply learning *where* a cortical parcel usually is. Importantly, we found that the optimal GAT model could identify three unique topologies for area 55b (typical, shifted, and split) and that predictions generated by our model replicated, with high fidelity, the same spatial organization patterns as identified in Glasser et al. (2016). This indicates that the model is capable of learning unique connectivity fingerprints of each cortical area on a subject-by-subject basis, rather than simply learning the group average fingerprint. As such, we do not believe that including the spatial prior in its current form inhibits the ability of the graph neural network models used in this analysis to identify atypical cortical topologies.

We compared three different graph neural networks: graph convolution networks, standard attention networks, and jumping-knowledge networks. We hypothesized that JKGAT networks would significantly outperform GAT networks due to the increased flexibility to learn optimized node-specific network depths. In their original formulation of the jumping-knowledge network architecture, Xu et al. (2018) found that including the jumping-knowledge mechanism improved model performance relative to the GAT in almost all of their comparisons. However, we found this not to be the case. This may be a consequence of the increased number of estimated parameters in the JKGAT networks, relative to the GAT—the jumping-knowledge aggregation layer learns the parameters for the aggregation function cells in addition to the attention head and projection matrix weights learned in the GAT networks. The lower classification accuracy at test time is possibly the result of model over-fitting, necessitating a larger training dataset. It is possible that the jumping-knowledge mechanism is generally more useful in the case where graph topologies vary considerably across a network, as opposed to more regular graphs such as cortical surface data.

As expected, network performance was dependent on both the size and duration of the training set, and duration of the testing data. Classification accuracy increased when models were trained on larger datasets consisting of shorter-duration images. Conversely, accuracy increased when models were deployed on longer-duration test data. It is important to note that we examined performance of our models on images of long scanning durations by concatenating multiple sessions together (30/60-min in the HCP, and 60/150/300-min in the MSC). It is unrealistic to expect study participants to be able to lay in an MRI scanner for single sessions of these lengths. However, it is useful to examine how model performance is impacted by tunable parameters like scan duration in order to best guide image acquisition in future studies. We found that utilizing repeated scans on individual subjects as independent training examples, rather than concatenating repeated scans together into single datasets, significantly improved our classification frameworks. This likely speaks to the ability of neural network models to generalize better to noise in the datasets. Training models on multiple samples of shorter-duration images more accurately captures the individual variability in the resting-state signal than fewer longer-duration images, thereby allowing the networks to more accurately learn a mapping between functional connectivity and cortical areal assignments.

Our methodology could be improved in a variety of ways. We chose not to perform intensive hyperparameter optimization, and instead focused our efforts on overall performance of the various network architectures as a function of network parameters and data parameters, and the applicability of trained models to new datasets. However, in the case where a classification model is meant to be distributed to the research community for open-source use, it would be prudent to perform a more extensive search over the best possible parameter choices.

The utility of functional connectivity has been shown in a variety of studies for delineating cortices (Blumensath et al., 2013; Arslan et al., 2015; Baldassano et al., 2015; Gordon et al., 2016). However, in recent years, using diffusion tractography for learning whole-brain cortical maps has been underutilized, relative to functional connectivity (Gorbach et al., 2011; Parisot et al., 2015; Bajada et al., 2017). Given cortical maps defined independently by tractography and functional connectivity, it is difficult to “match” cortical areas across maps to compare biological properties, so heuristics are often applied. Few studies have simultaneously combined functional connectivity and tractography to better inform the prediction of cortical maps. Recent work has extended the idea of variational auto-encoders to the case of multi-modal data by training coupled auto-encoders to jointly learn embeddings of multiple data types. In Gala et al. (2021), the authors apply this approach to jointly learn embeddings defined by transcriptomics and electrophysiology that allow them to identify cell clusters with both similar transcriptomic and electrophysiology properties. Future work could apply similar ideas to aggregate functional and diffusion-based connectivity signals.

The majority of recent studies have approached the cortical mapping problem from the perspective of generating new parcellations from underlying neurobiological data using unsupervised clustering or spatial gradient methods. These approaches attempt to delineate areal boundaries by grouping cortical voxels together on the basis of similarity between their features. Spatial gradient-based methods explicitly define areal boundaries, while clustering methods define these boundaries implicitly. However, both approaches are distinct from methods that utilize pre-existing or pre-computed parcellations as templates for mapping *new* data. In the current analysis, we were concerned with the latter problem.

Clustering and spatial gradient approaches are often interested in relating newly-generated cortical maps to underlying *in vitro* measures, such as transcriptomics or cytoarchitectural results. Clearly, it is impossible to acquire this data in human subjects simultaneously with *in vivo* data. Various projects have attempted to build cytoarchitectural datasets from post-mortem subjects to use as a basis of comparisons for maps generated *in vivo* (Amunts et al., 2020). While some cortical areas have been recapitulated using both *in vitro* and *in vivo* features, this is not a general rule across the cortex. As such, cross-modal verification is often difficult, and leaves room for methods and datasets than can improve upon the validation of cortical mapping studies.

One limitation of our analysis concerns the use of different versions of the multi-modal surface matching for cortical surface alignment for the S500 HCP data release (Glasser et al., 2013; Robinson et al., 2014), the S1200 release (Robinson et al., 2018), and for the subject-level HCP-MMP parcellations (Glasser et al., 2016), which used a different regularization term. These differences between the three registration methods result in a slight spatial misalignment between the training labels and the cortical features. While the S500 data release utilized MSMSulc, a spherical surface registration driven by cortical folding patterns, the S1200 release utilized MSMAll, and incorporated functional connectivity into the spatial resampling step. Glasser et al. (2016) used a prototypical version of MSMAll in addition to MSMSulc, and thereby incorporated additional features derived from resting-state networks to drive the surface matching process. Importantly, this discrepancy between the training labels and training features is not a flaw in our methodology itself, and correcting for this difference in the registration approach would only improve the results of our analysis. As we showed in Figure 10, incorporating MSMAll-processed data from the S1200 dataset, instead of MSMSulc-processed data from the S500 dataset, improved model classification accuracy by nearly 5%. We hypothesize that this improvement would only increase if we had access to the data processed with the prototypical version of MSMAll. Based on the comparisons of subject-level predictions with the subject-level ground truth MMP maps, our models performed well in spite of these registration discrepancies. Our results lend evidence to the robustness of graph neural networks for learning cortical maps from functional connectivity.

Finally, participants in both the HCP and MSC studies were healthy young adults, and the datasets had been extensively quality controlled. Little to no work has been done on extending connectivity-based classifiers to atypical populations, such as to individuals with neurodegeneration. It is unknown how a model trained on connectivity properties from healthy individuals would perform in populations where connectivity is known to degrade. While our model (and that developed by Glasser et al., 2016) predict maps based on healthy individuals, it is possible that some studies would need to train population-specific models.

## Data Availability Statement

The original contributions presented in the study are included in the article/Supplementary Material, further inquiries can be directed to the corresponding author/s.

## Author Contributions

KE conceptualized this study, developed the code, performed the analyses, and wrote the bulk of the document. TG provided comments and neuroscientific insight into the analysis, contributed to the editing, and organization structure of the manuscript. DH provided extensive neuroscientific and technical guidance for this work, contributed to the editing, and organizational structure of the manuscript. All authors contributed to the article and approved the submitted version.

## Funding

This project was supported by grant NSF BCS 1734430, titled Collaborative Research: Relationship of Cortical Field Anatomy to Network Vulnerability and Behavior (TG, PI).

## Conflict of Interest

The authors declare that the research was conducted in the absence of any commercial or financial relationships that could be construed as a potential conflict of interest.

## Publisher's Note

All claims expressed in this article are solely those of the authors and do not necessarily represent those of their affiliated organizations, or those of the publisher, the editors and the reviewers. Any product that may be evaluated in this article, or claim that may be made by its manufacturer, is not guaranteed or endorsed by the publisher.
