# Supplementary material for: Learning Cortical Parcellations Using Graph Neural Networks
Source: Front Neurosci. 2021 Dec 24;15:797500. doi: 10.3389/fnins.2021.797500 (PMC8739886; doi:10.3389/fnins.2021.797500)
Supplement: Supplementary file 1 [file Data_Sheet_1.pdf]

Supplementary Material

1 PERFORMANCE IS DEPENDENT ON NETWORK AND IMAGING PARAMETERS

1.1 Standard error estimates of classification accuracy

Table S1. Standard error estimates of model classification accuracy as a function of network architecture and parameterization.

		Model			
Parameter	Value	Baseline	GCN	GAT	JKGAT
Network Depth	3	0.24%	0.21%	0.21%	0.21%
	6	0.25%	0.22%	0.20%	0.20%
	9	0.26%	0.21%	0.20%	0.20%
Hidden Channels	16	0.23%	0.21%	0.21%	0.21%
	32	0.24%	0.21%	0.21%	0.21%
	64	0.23%	0.22%	0.20%	0.21%
Dropout Rate	0.1	0.24%	0.21%	0.21%	0.21%
	0.3	0.19%	0.21%	0.21%	0.21%
	0.5	0.17%	0.20%	0.19%	0.20%
	0.7	0.16%	0.21%	0.18%	0.19%
Attention Heads	4			0.21%	0.21%
	8			0.21%	0.21%
	12			0.20%	0.21%
Aggregation Function	concat				0.21%
	lstm				0.21%

We used a single hold-out dataset to estimate final performance metrics for all models. In order to better understand the variability in the accuracy estimates as a function of resampling of the test data, we estimated bootstrapped standard errors of the mean classification accuracy (Table S1). From the 148 test subjects, we sampled 100 subjects with replacement and compute the mean classification accuracy for this sub-sample. We repeated this process for 1000 iterations, and report the standard deviation of these mean estimates as the standard error of model accuracy. We found that the standard error was lower in the graph neural networks relative to the baseline neural network, indicating that predictions generated by the more flexible models are more consistent, irrespective of resampling of the test data. There was little variability

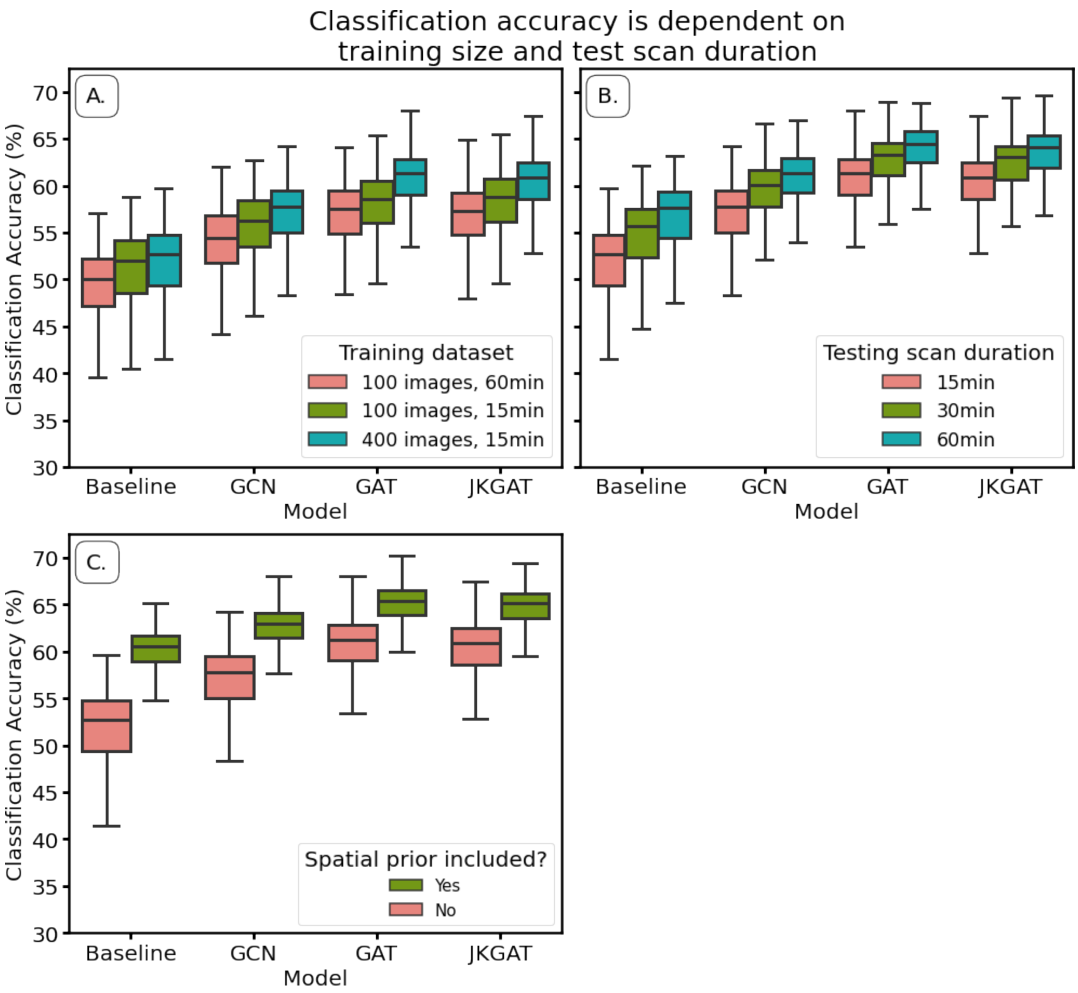

Figure S1: Classification accuracy rates of each model using the regionalized connectivity features, and the default model parameters, as a function of training dataset and testing image acquisition parameters. Classification accuracy improved with increasing training dataset set size (**A**) and increasing test image duration (**B**). Accuracy improved when including the spatial prior (**C**).

in the model standard errors as a function of the network architecture parameters, except for the dropout parameter. Standard errors decrease as dropout rates increased. However, classification accuracy decreased as dropout rates increased (see main document).

We found that, when fixing the training dataset duration and size, longer test image durations yield increased classification accuracy (Figure S1). However, accuracy was dependent on the interaction between training image duration and testing image duration. When fixing the training dataset size, models trained on 15-minute data performed better when tested on 15-minute data, while models trained on 60-minute data performed better when tested on 60-minute data (Table S2).

1.2 Error rates are highest near region boundaries

Predicted parcellations (Figure S2) and prediction error maps (Figure S3) generated by each default neural network model (with a spatial prior) are illustrated for a single HCP subject (subject 100408). Graph neural network models generated parcel predictions that were more contiguous with very few isolated vertex clusters. In contrast, the baseline model, which does not take into account adjacency information, generated parcels that were very noisy near the parcel boundaries, even when including the spatial prior. This indicates that the graph neural networks are able to learn embeddings that better distinguish between adjacent cortical areas, relative to performance of the baseline network, and points to a strength of incorporating adjacency information into the classification procedure.

Table S2. Accuracy estimates for predictions generated by the optimal model architecture. We found that model accuracy was dependent on training and testing image duration.

		Testing Duration		
		15min	30min	60min
Training Dataset	100 images, 60min	56.89%	60.17%	62.23%
	100 images, 15min	57.88%	60.29%	61.77%
	400 images, 15min	60.56%	62.58%	63.75%

We examined how the spatial distribution of misclassified vertices was related to distance of those errors from cortical areal peripheries. If our models are performing well, the majority of misclassified vertices should exist at the region boundaries. Given that region boundaries are areas of transition between cortical signals, like functional or structural connectivity, vertices at region boundaries are more difficult to classify than vertices in region cores. Vertices closer to region boundaries are more likely to combine signals from adjacent cortical regions as network depth increases.

Supplementary Figure S4 demonstrates that the majority of classification errors occur at the region boundaries, and that the likelihood of misclassification decreases further away from region boundaries. We found that roughly 50%, 30%, and 12% of all misclassified vertices occur at a distance of 0, 1, and 2 hops away from region boundaries. That is to say, nearly half of all misclassifications occurred where two ”true” regions meet. Also shown is the fraction of *all* vertices occurring within k hops from ”true” region borders (dashed black lines). This plot illustrates that the fraction of all vertices at a distance of k hops from the border was considerably less than the fraction of errors occurring at a distance of k hops from the border, indicating that the majority of errors are restricted to region boundaries. We found that, of all possible cortical vertices, 33% existed at a region boundary, while roughly 50% of classification errors occurred at a region boundary.

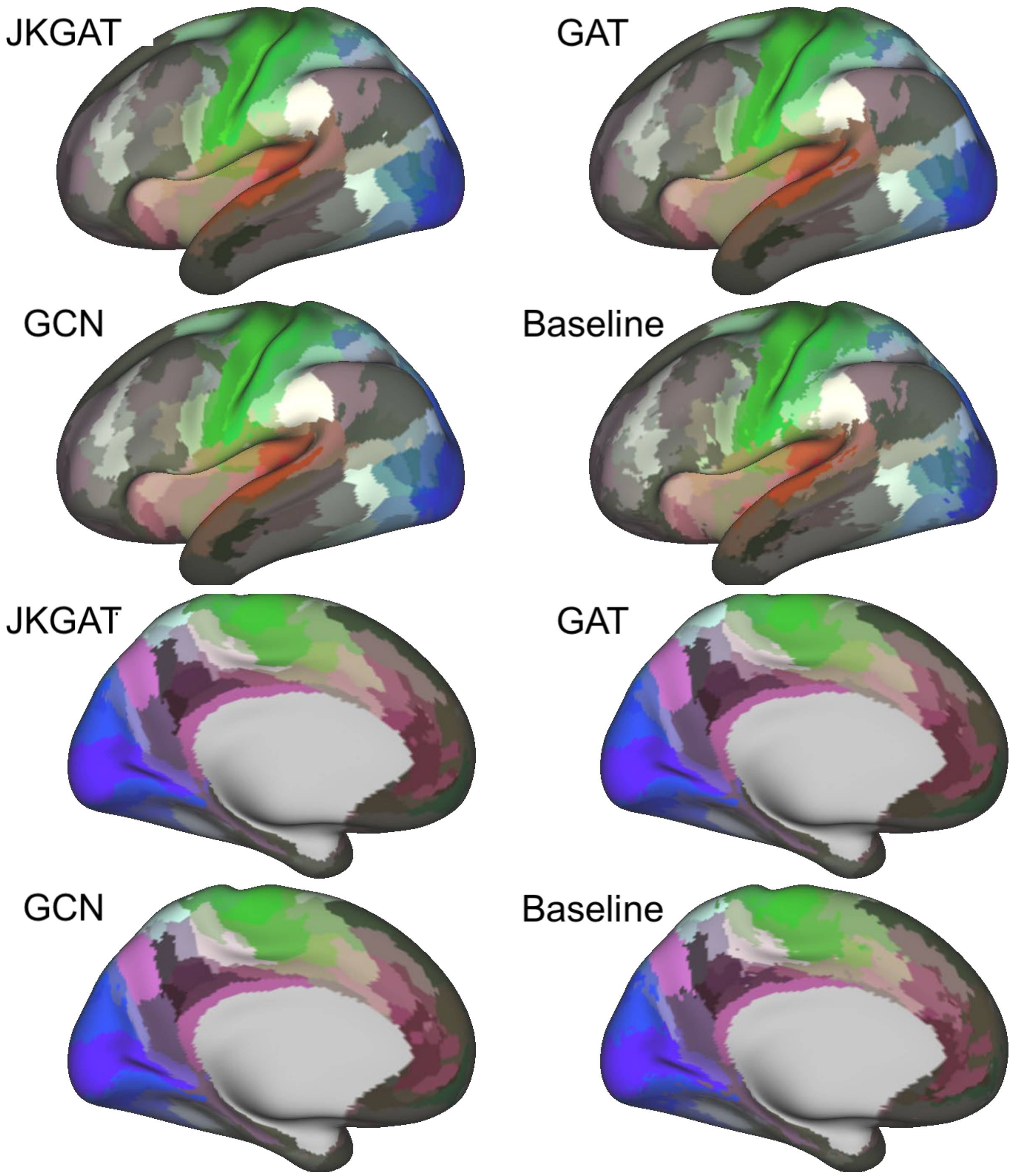

Figure S2: Predictions generated by each model, using the default model parameters, when trained/tested on the regionalized connectivity features.

2 PREDICTION CORRESPONDENCE WITH THE GROUND TRUTH AND GROUP-AVERAGE MAPS

2.1 Correspondence of predicted maps with the HCP-MMP atlas

Only the HCP data has underlying ground truth maps against which we could compare our predicted parcellations. However, because the MSC dataset is also in spatial correspondence with the *fsaverage_LR32k* map, we computed the correspondence of maps predicted on the MSC subjects with the HCP-MMP atlas

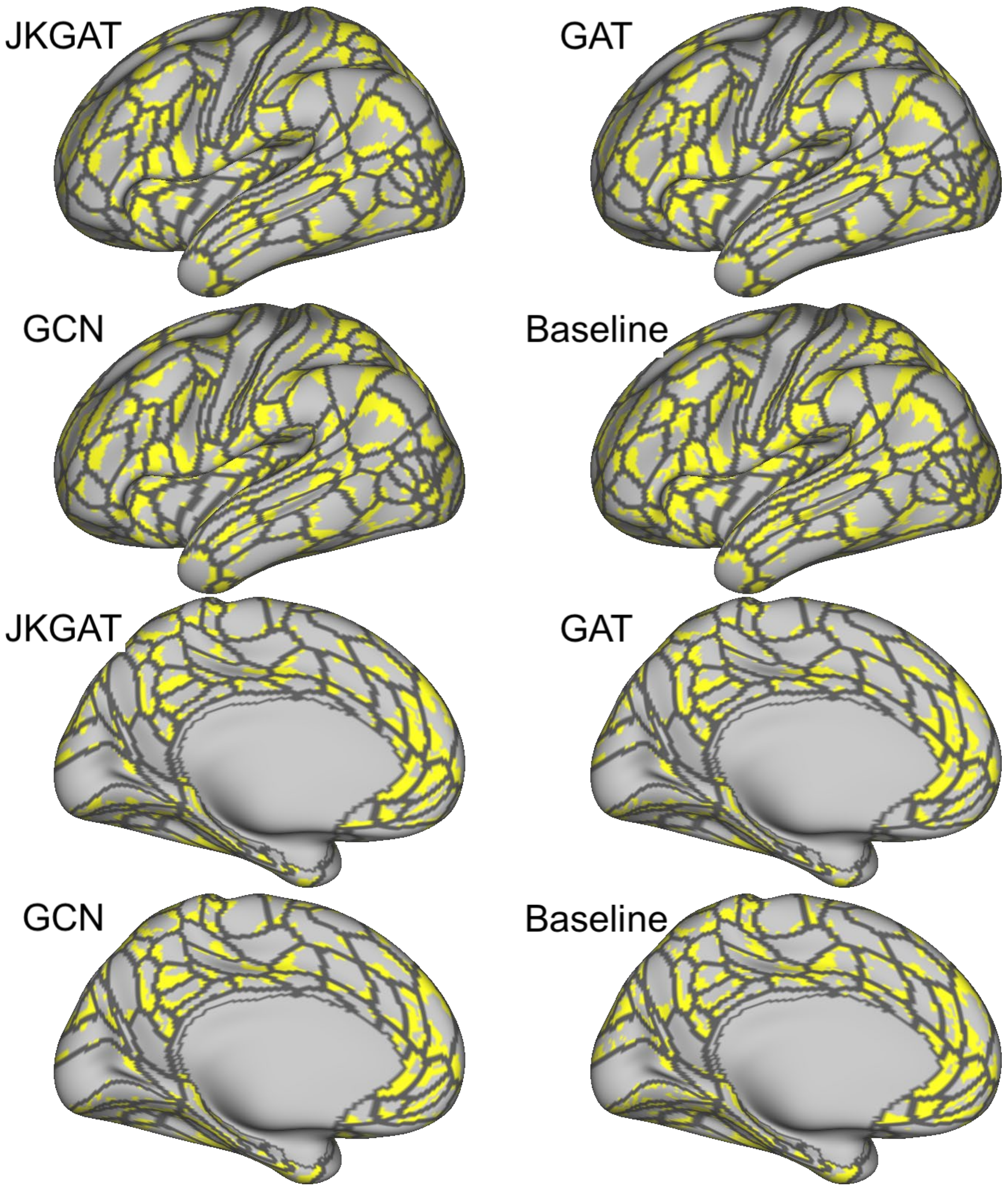

Figure S3: Prediction errors generated by each model, using the default model parameters, when trained/tested on the regionalized connectivity features.

(the average of the ground-truth maps) in order to determine how our models perform on data that does not have an underlying ground truth map.

Correspondence of predictions computed on the MSC dataset with the HCP-MMP atlas was slightly less (65.39%, 68.03%, 69.58%, 70.04% in the 30-, 60-, 150-, and 300-minute datasets) than correspondence of those predictions computed on the HCP dataset (81.70%, 83.49%, 84.35% in the 15-, 30-, and 60-minute datasets) (Figure S5). Relative to correspondence with the ground truth maps, HCP maps correspondence

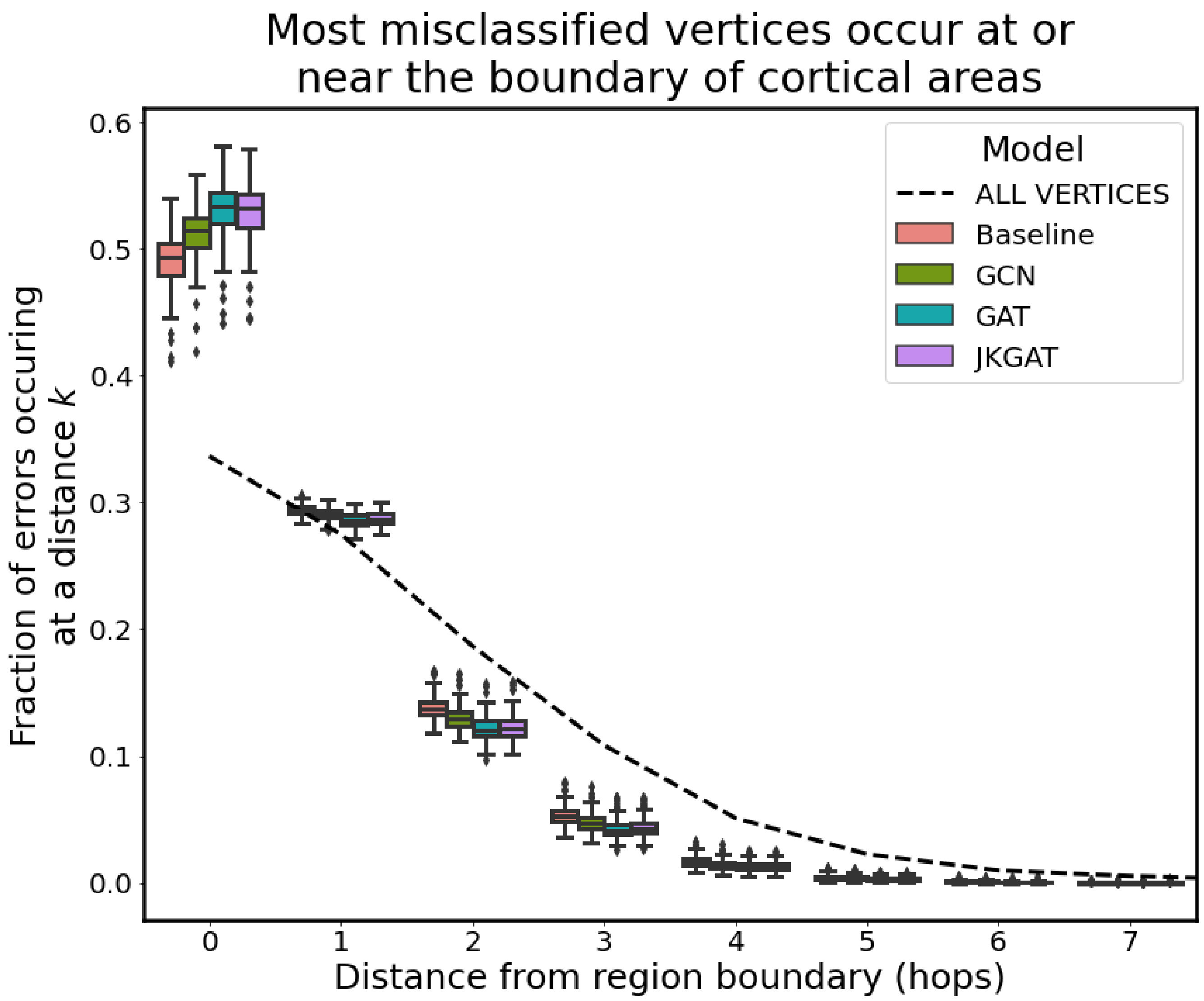

Figure S4: The majority of misclassified vertices occurred at or near areal boundaries. Fraction of misclassified vertices at k hops from cortical areal boundaries. These results are produced using our default models with 3 layers, 32 hidden channels, a dropout rate of 0.1, 4 attention heads, and an LSTM aggregation function. The dashed black line represents the fraction of **all** cortical vertices that exist at a distance k of cortical areal boundaries.

better with the atlas by upwards of 4% using the default models. Correspondence of predictions with the HCP-MMP increased as the testing scan duration increased and when including a spatial prior.

2.2 Within versus between subject prediction correspondence

In Figure S6, we illustrated between-subject prediction correspondence, and correspondence of subjects' predictions with their ground-truth counterparts and with the HCP-MMP atlas using predictions computed from the optimal model. When comparing predictions between two different subjects, the subject-level predictions are less similar to one another (74.89%, 77.46%, 78.93%) than either subject's prediction is to the corresponding ground truth map (77.49%, 78.95%, 79.91%), or to the HCP-MMP atlas (81.70%, 83.49%, 84.35%) for 15/30/60-minute test-durations. Predictions were more similar to the HCP-MMP atlas than they were to the ground-truth maps, and least similar to predictions generated on other subjects. Overall, similarity between any two parcellations (prediction, truth, or atlas) increased with increasing test-scan duration. Likewise, repeated scans from the same subject showed high correspondence between predictions

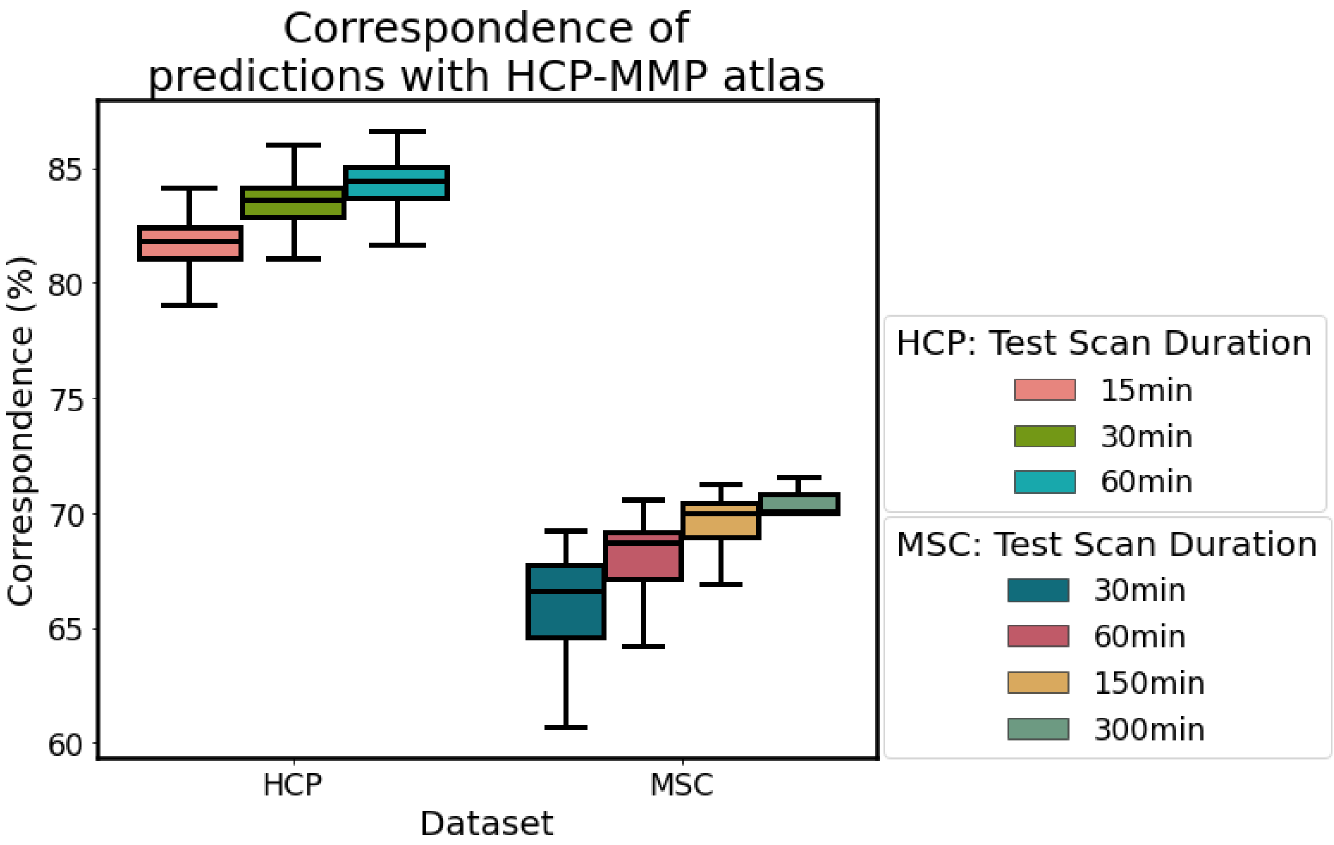

Figure S5: Correspondence of parcellations predicted by the optimal model, as a function of test scan duration for HCP (left) and MSC (right) data.

from the same scanning duration (Dice coefficients of 0.81 and 0.86 for 15-minute and 30-minute scanning durations; see main document).

3 GRAPH NEURAL NETWORKS LEARN SUBJECT-SPECIFIC PARCELLATION TOPOLOGIES

3.1 Exemplar areal predictions overlap with histological features

We first show exemplar subject-level parcel predictions to illustrate how these predictions relate to the group-level areal boundaries and the subject-level histological features. Model areal probabilities were restricted to contiguous local regions – that is, non-zero probabilities are restricted to limited areas of the cortex. High-probability areas closely followed the consensus areal boundaries, as did the final network predictions, but also capture subject specific organization.

The following figures show the network probabilities (top row), final discrete network predictions (second row), cortical thickness (third row), and myelin content (bottom row), for a series of cortical areas for three HCP subjects: 100408 (left column), 102311 (middle column), and 105216 (right column). In each subplot, in dark blue, we show the consensus level areal boundary for each cortical area. Probabilities are thresholded at 0.005, the probability of uniformly assigning a vertex to a random label from the 180 possible cortical areas. We show plots for area V1 (Figure S7), MST (Figure S8), 4 (Figure S9), and PEF (Figure S10).

3.2 Graph neural network learn unique topologies of area 55b

We examined how well graph neural networks learned subject-specific topologies for area 55b. Area 55b was identified by Glasser et al. (2016) as having three distinct topologies, relative to the group-average 55b areal boundaries, which the authors referred to as typical, shifted (where the subject-level 55b prediction

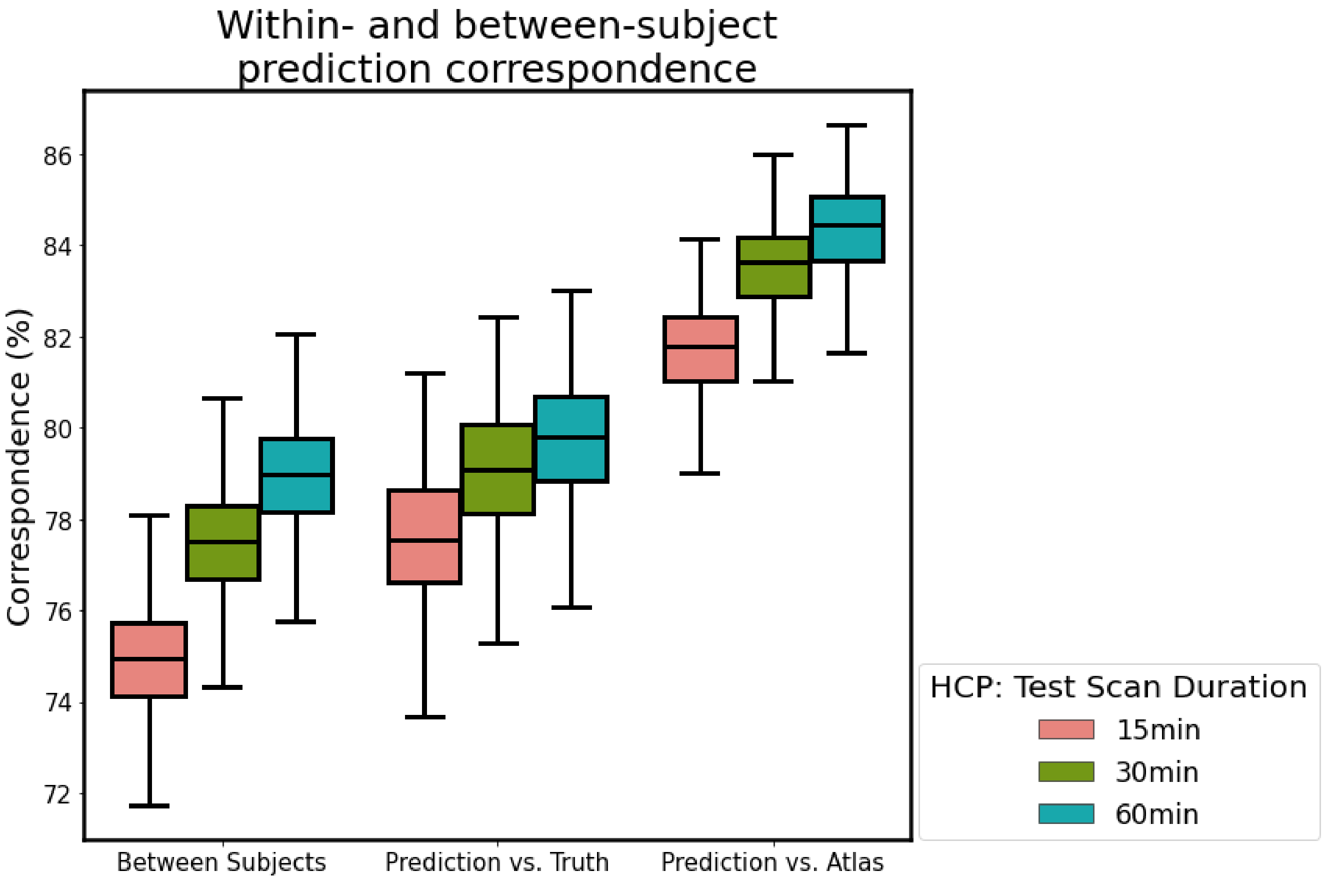

Figure S6: Between-subject prediction correspondence (left), and correspondence of predictions with their corresponding ground truth maps (middle) and with the HCP-MMP atlas (right), as a function of test-scan duration.

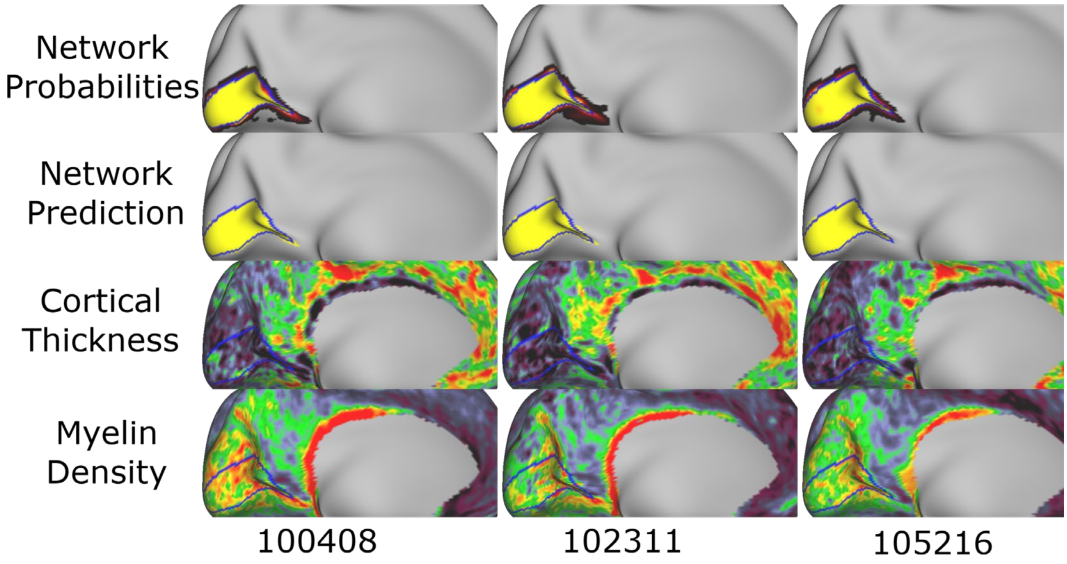

Figure S7: Network probabilities (row 1), final discrete network output (row 2), cortical thickness (row 3), and myelin content (row 4) for area V1 for three HCP subjects. We show the consensus areal boundary in dark blue.

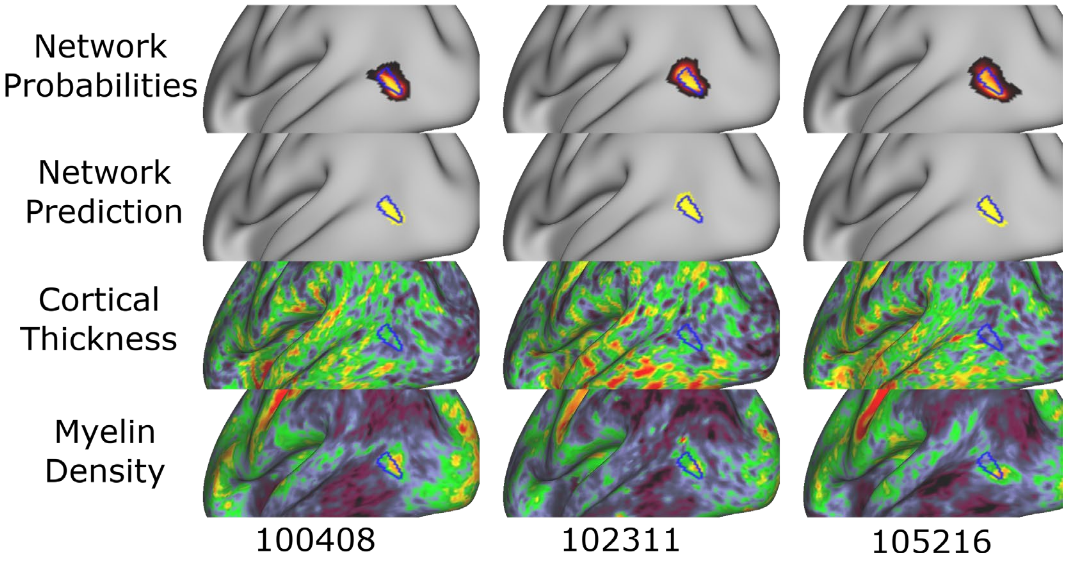

Figure S8: Network probabilities (row 1), final discrete network output (row 2), cortical thickness (row 3), and myelin content (row 4) for area MST for three HCP subjects. We show the consensus areal boundary in dark blue.

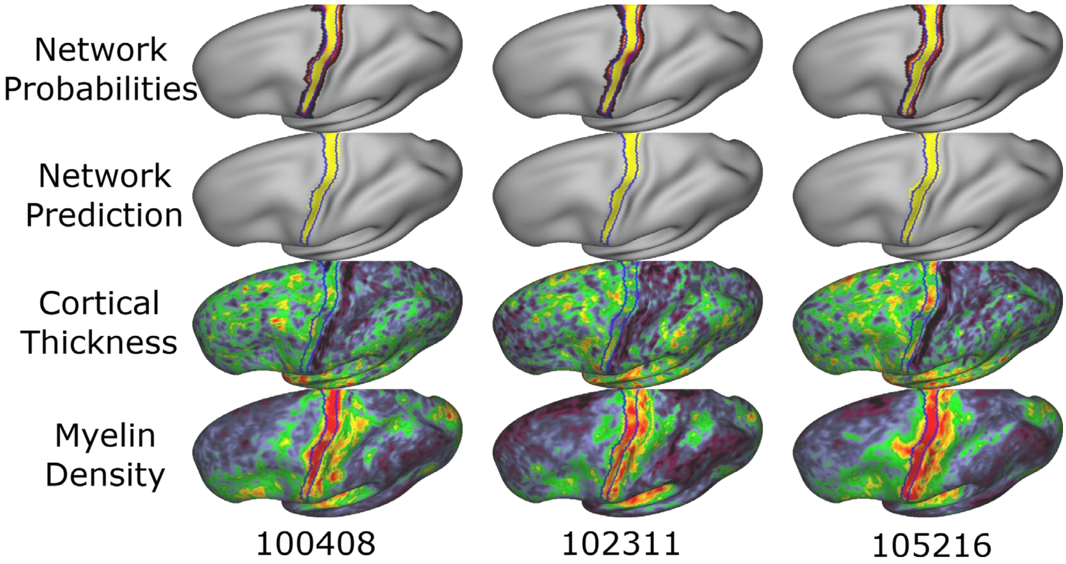

Figure S9: Network probabilities (row 1), final discrete network output (row 2), cortical thickness (row 3), and myelin content (row 4) for area 4 for three HCP subjects. We show the consensus areal boundary in dark blue.

was translated across the cortex, relative to the group average boundary), and split (where the subject-level 55b prediction was split into multiple contiguous subregions).

We found that GNN models learned subject-specific parcellations that vary considerably from the group-average parcellation, again indicating that the model is not simply learning *where* a parcel is, but rather learning a unique connectivity fingerprint of the specific cortical area. The optimal model was able to accurately learn the subject-specific topologies of area 55b for all three layouts (typical, shifted, split). These predictions accurately recapitulated the topologies identified by Glasser et al. (2016).

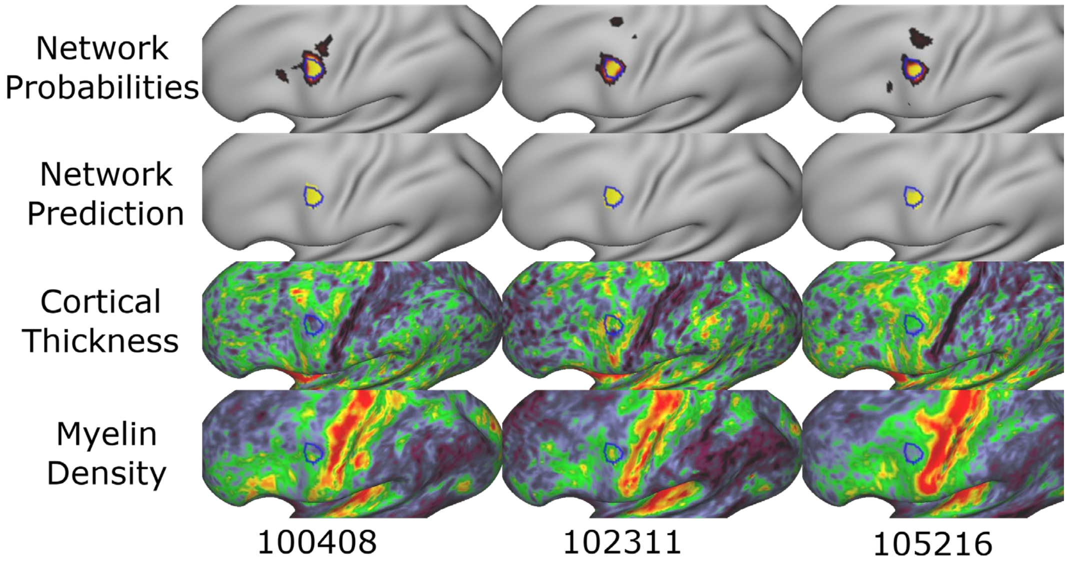

Figure S10: Network probabilities (row 1), final discrete network output (row 2), cortical thickness (row 3), and myelin content (row 4) for each PEF for three HCP subjects. We show the consensus areal boundary in dark blue.

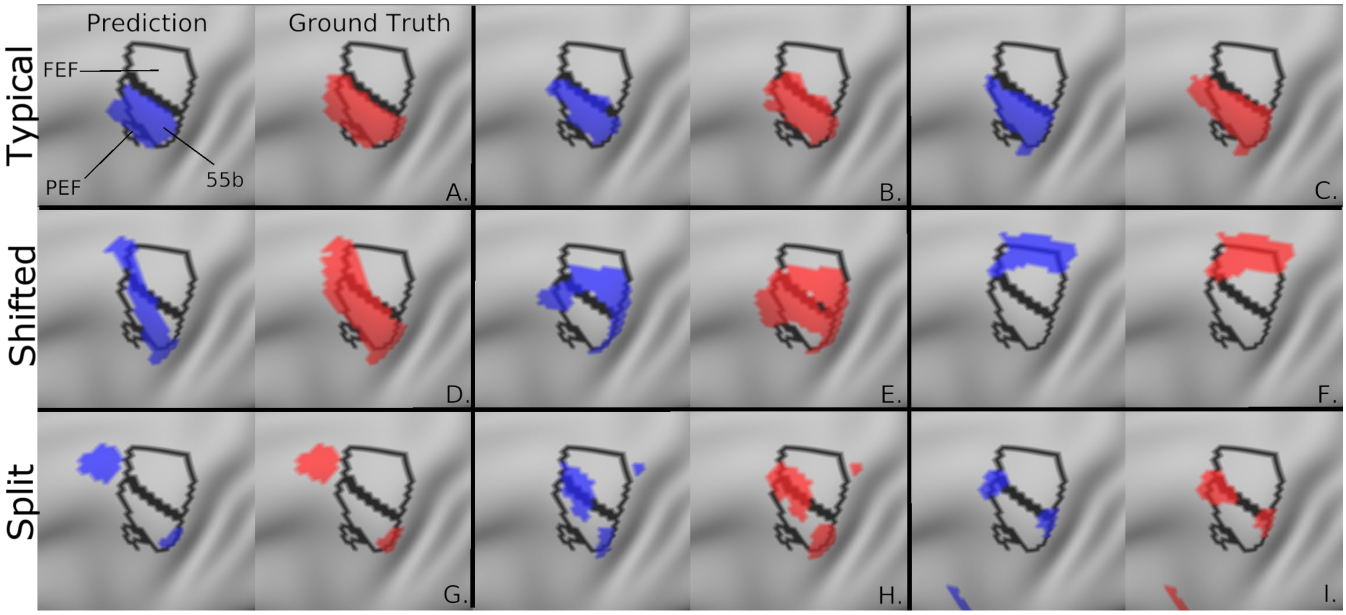

Figure S11: Exemplar topologies for area 55b. Glasser et al. (2016) identified ”typical” (top), ”shifted” (middle), and ”split” (bottom) topologies. The group-level areal boundaries are shown in black for area FEF (superior), 55b (middle), and PEF (inferior). ”Ground truth” predictions generated by (Glasser et al., 2016) are shown in red.

We next examined how reproducible these 55b topologies were for a set of subjects. Figure S12 illustrates predictions for area 55b for 6 independent HCP subjects computed using the optimal model from each of the four 15-minute independent resting-state sessions. Just as in Figure S11, predictions across sessions respected the general placement of the group-average areal boundary, but differed in their subject-level topologies. These topologies were reproducible within subjects, indicating that the graph neural networks, again, are able to learn subject-specific connectivity fingerprints and that these fingerprints are conserved

across scanning sessions. Notably, for subject 114924, we were able to identify two contiguous components for area 55b that are quite consistent spatially across scanning sessions. Similarly, for subject 133928, we found that the subject-level predictions deviated considerably from the group-average areal boundary, and that the shape and placement of this deviation was remarkably consistent across the four sessions. We were again able to identify the typical (100408, 109325, 133928), shifted (128632, 130013), and split (114924) topologies for this set of exemplar subjects.

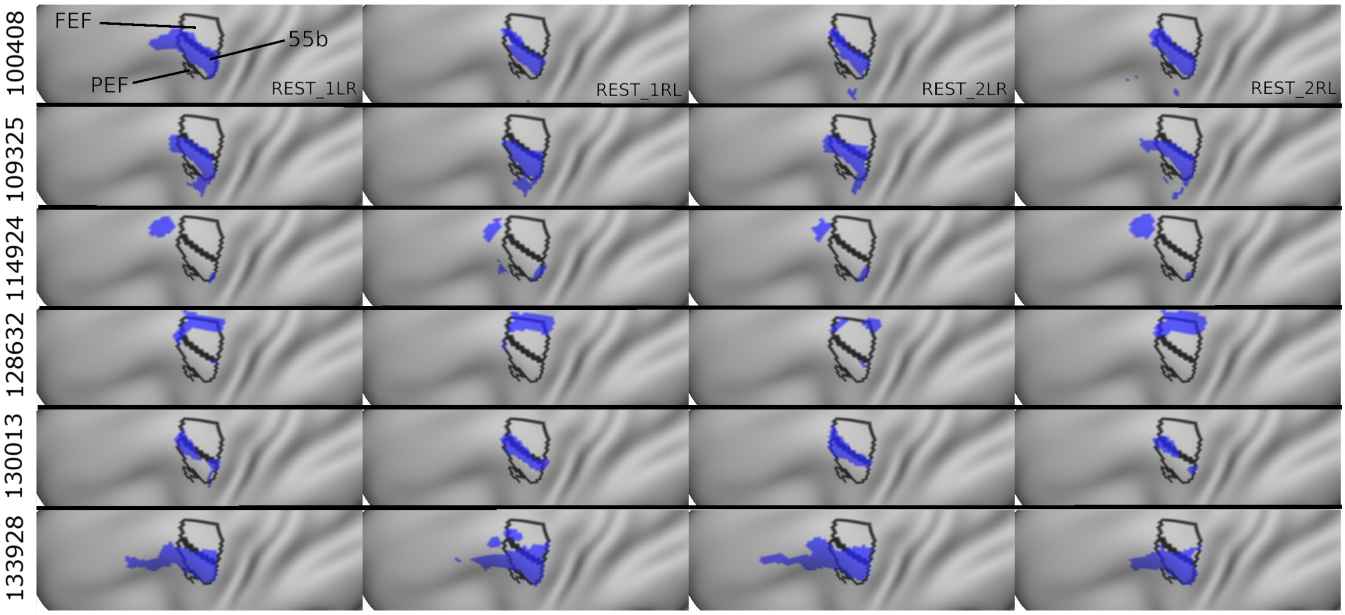

Figure S12: Reproducibility of subject-level topologies for area 55b for 6 exemplar subjects across each of the 4 independent resting-state sessions. The group-level areal boundaries are shown in black for area FEF (superior), 55b (middle), and PEF (inferior).

3.3 Training and validation dataset performance during model training

In Figure S13, we show model performance for the optimal model architecture, trained on the resting-state network datasets, for the training and validation datasets as a function of training epoch. As a reminder, we used the cross entropy loss as implemented in PyTorch. We trained using mini-batches of size $s = 10$ graphs and accumulated the gradient from all batches, prior to computing the gradient update. Model performance on the training and validation datasets are quite comparable over the duration of model training. Validation loss is less than training loss until epoch 226. The optimal model occurs at epoch 689, where validation loss is minimized.

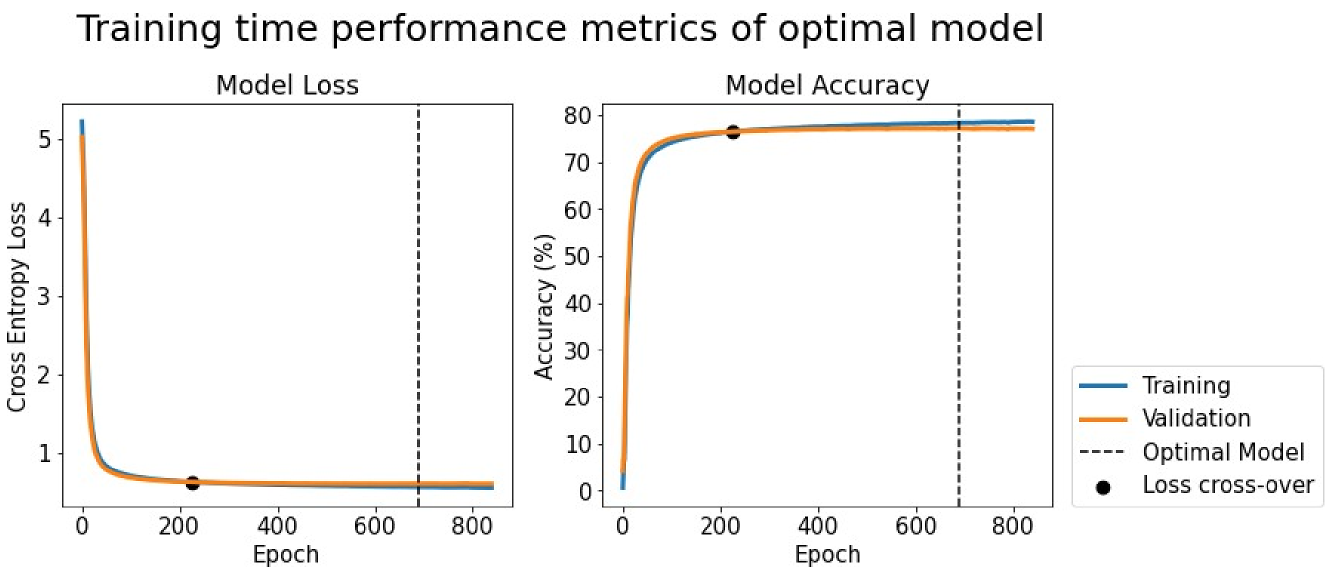

Figure S13: Model performance as a function of training epoch, where training is indicated in blue, and validation is indicated in orange. We show training and validation loss (left) and accuracy (right). The optimal model is indicated by the dashed line, the point at which validation loss is minimized. We also identify the point at which training loss becomes less than validation loss using with a black dot.

REFERENCES

Glasser, M. F., Coalson, T. S., Robinson, E. C., Hacker, C. D., Harwell, J., Yacoub, E., et al. (2016). A multi-modal parcellation of human cerebral cortex. *Nature* 536, 171–178. doi:10.1038/nature18933
